# Bridging the gap between postembryonic cell lineages and identified embryonic neuroblasts in the ventral nerve cord of *Drosophila melanogaster*

**DOI:** 10.1242/bio.201411072

**Published:** 2015-03-27

**Authors:** Oliver Birkholz, Christof Rickert, Julia Nowak, Ivo C. Coban, Gerhard M. Technau

**Affiliations:** Institute of Genetics, University of Mainz, D-55099 Mainz, Germany

**Keywords:** CNS development, Neuroblast, Cell lineage, Flybow, Segmental patterning, *Drosophila*

## Abstract

The clarification of complete cell lineages, which are produced by specific stem cells, is fundamental for understanding mechanisms, controlling the generation of cell diversity and patterning in an emerging tissue. In the developing Central Nervous System (CNS) of *Drosophila*, neural stem cells (neuroblasts) exhibit two periods of proliferation: During embryogenesis they produce primary lineages, which form the larval CNS. After a phase of mitotic quiescence, a subpopulation of them resumes proliferation in the larva to give rise to secondary lineages that build up the CNS of the adult fly. Within the ventral nerve cord (VNC) detailed descriptions exist for both primary and secondary lineages. However, while primary lineages have been linked to identified neuroblasts, the assignment of secondary lineages has so far been hampered by technical limitations. Therefore, primary and secondary neural lineages co-existed as isolated model systems. Here we provide the missing link between the two systems for all lineages in the thoracic and abdominal neuromeres. Using the Flybow technique, embryonic neuroblasts were identified by their characteristic and unique lineages in the living embryo and their further development was traced into the late larval stage. This comprehensive analysis provides the first complete view of which embryonic neuroblasts are postembryonically reactivated along the anterior/posterior-axis of the VNC, and reveals the relationship between projection patterns of primary and secondary sublineages.

## Introduction

Neural stem cells give rise to typical sets of daughter cells, known as lineages or cell clones, which often comprise many different cell types. The identification and precise description of these lineages is the basis to both understanding how cellular diversity and patterning is achieved in the developing CNS and how the individual lineages (developmental units) contribute to the establishment of neural circuits (functional units).

In the fruitfly *Drosophila melanogaster* almost all neural stem cells (called neuroblasts, NBs) have been individually described in the brain (Urbach et al., 2003; Urbach and Technau, 2003) and the VNC (Birkholz et al., 2013; Broadus et al., 1995; Doe, 1992). Compared to the complex composition of the brain, which is set up by approximately 100 NBs per hemisphere, the VNC appears relatively simple, being composed of repetitive segmental units called neuromeres which facilitates analysis at the level of identified cells. Each hemineuromere is produced by about 30 NBs (Doe, 1992), with the exception of the gnathal and the most terminal ones which comprise a reduced NB-set (Birkholz et al., 2013) (Rolf Urbach, unpublished results). The primary cell lineages that are generated by these NBs during embryogenesis are diverse and provide the functional nervous system of the larva (reviewed by Hartenstein et al., 2008). All embryonic cell clones of the VNC have been described in detail and each of them is rather invariant, unique and characteristic for a specific NB (Bossing and Technau, 1994; Bossing et al., 1996b; Schmid et al., 1999; Schmidt et al., 1997).

During postembryonic development, *Drosophila* undergoes dramatic morphological changes towards sculpting the adult fly which exhibits a much more complex behavioural repertoire compared to the larva. Accordingly, significant remodelling of the CNS occurs during larval and pupal development and a vast number of additional cells are generated during a second wave of proliferation. These postembryonic progeny cells (secondary lineages) have the same origin as the primary lineages, and are thus part of the same NB clones (Prokop and Technau, 1991). After a period of mitotic dormancy, a specific subpopulation of NBs becomes reactivated and resumes proliferation in the larva (reviewed by Maurange and Gould, 2005). In the VNC the number and mitotic activity of these NBs is segment-specific (Truman and Bate, 1988) and already determined in the embryonic neuroectoderm (Prokop et al., 1998). Approximately 23 (+1 unpaired) NBs per hemineuromere are reactivated in thoracic segments T1–T3, about 12 (+ 1 unpaired) NBs in the first abdominal segment (A1), 4 NBs in A2 and 3 NBs in A3–A7 (Truman and Bate, 1988), while the remaining NBs undergo apoptosis at the end of embryogenesis (Peterson et al., 2002). The time window of postembryonic proliferation is also segment-specific (Truman and Bate, 1988) and is terminated in thoracic segments by a final division and differentiation of NBs (Maurange et al., 2008) and in segments A3–A7 by programmed cell death (PCD) triggered via a pulse of *Hox*-gene expression (Bello et al., 2003).

The secondary lineages are arranged in clusters of hemilineages (Lin et al., 2010; Truman et al., 2010) that form short, unbranched fascicles and their individual marker gene expression and morphology have been described in detail (Kuert et al., 2014; Lacin et al., 2014b; Truman et al., 2004). They undergo maturation during metamorphosis, while the primary cells already reveal complex dendritic arborisations in the larva (reviewed by Hartenstein et al., 2008).

Although detailed descriptions exist for both primary and secondary lineages in the VNC, it has so far, for technical reasons, not been possible to link the two parts. On the one hand, the tracers used for detection of primary clones (DiI; HRP) are diluted to undetectable levels in secondary lineages (e.g. Prokop and Technau, 1991). On the other hand, mosaic analysis with a repressible cell marker (MARCM), which was used to uncover secondary lineages, does not label embryonic lineages as the perdurance of Gal80 prevents their visualisation (Lee and Luo, 1999). Although attempts have been made to relate some embryonic and postembryonic cell lineages using the morphology of characteristic embryonic cells and marker gene expression in late larvae (Truman et al., 2004), the existing evidence is rather indirect and incomplete.

**Fig. 1.**
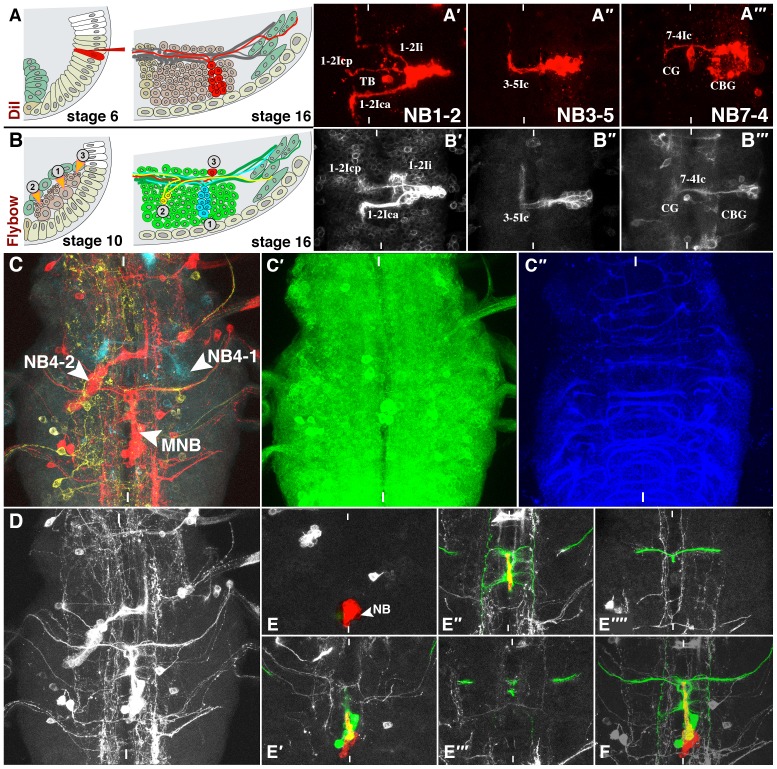
Generation and Processing of Clonal Data. (A–B′″) Lineage tracing using DiI labelling (Bossing et al., 1996b; Schmidt et al., 1997; A–A′″) as compared to applying Flybow 1.1B (Shimosako et al., 2014; B–B′″). (A) Schematic transverse sections showing right ventral half of a stage 6 and a stage 16 embryo; ventral is down, mesoderm in mint, mesectoderm and neuroectoderm are in beige. Classical labelling of single neuroectodermal progenitor cells (stage 6, capillary with red dye from right) allows targeted application of the dye (e.g. dorsal versus ventral cells or thoracic versus abdominal segments) and results in complete lineages at the end of embryogenesis (stage 16). (B) Schematic transverse sections showing right ventral half of a stage 10 and a stage 16 embryo (*elav*>*Flybow 1.1B*). Independent recombination events in (1) a NB, (2) a ganglion mother cell and (3) a postmitotic neuron, caused by a heatshock driven flipase are symbolised with arrowheads (stage 10). In stage 16 these events result in multicellular (1), two cell (2) or single cell clones (3), respectively, labelled randomly by mCherry-, mCitrine- or mTurquoise-expression. Maximum clone size and -density can be controlled by the timepoint and strength of the heatshock, but clone locations are arbitrary. Corresponding clonal types labelled by both methods can be unambiguously identified *in vivo* based on typical morphological characteristics: compare (A′–A′″) and (B′–B′″) respectively (dorsal views of early stage 17 VNCs). CBG, cell body glia; CG, channel glia; Ic, Ica, Icp, Ii, TB are specific interneurons (see Bossing et al., 1996b; Schmidt et al., 1997). (C–F) Demonstration of the Flybow 1.1B approach used to record, process and present the larval clone data. (C,D) Dorsal views on thoracic segments of a late L3 larva, representing the unprocessed stack that was used for Fig. 3B. (C′) resembles the default state (GFP-expression) in all neuronal cells without recombination event, while (C) extracts the three channels resulting from recombination. The yellow channel reveals only single cell clones, red shows a MNB- and a NB4-2 clone, cyan a NB4-1 clone. (C″) Nrt-antibody staining, visualising commissural fibres, that served as landmarks to identify secondary lineages (see text and Fig. 2 for details). (D) If a clone was chosen for presentation, the respective channel (here: the red one) was transformed into grey. Then every single layer was inspected and secondary cells and their projections were coloured red. Primary cells and all remaining projections clearly connected to the clone were coloured green (for discrimination between primary and secondary cells see text). (E–E″″) Stacks of several images from ventral (E) to dorsal (E″″) positions, documenting the procedure that results in (F), i.e. the complete stack and the same image as shown in Fig. 3B. White vertical bars indicate the midline.

To bridge the gap between the embryonic and postembryonic lineage model systems, we established an alternative approach: By adapting the Flybow system (Hadjieconomou et al., 2011; Shimosako et al., 2014) we were able to label primary lineages in a way that allowed their identification and documentation in the VNC of living embryos (Fig. 1A,B). Upon determining the identity of the lineages, the specimens were allowed to develop until the late third larval instar (L3), when the composition of the entire lineages was analysed (Fig. 1C–F). As the primary lineages have been previously linked to identified NBs, we established which of these NBs are reactivated in the larva and which are not. Furthermore, we documented the relationship between the projection patterns of primary and secondary sublineages. Finally, as we identified the embryonic precursor of all postembryonic subclones in each of the thoracic and abdominal neuromeres, segment-specific differences among serially homologous lineages have been disclosed. This comprehensive analysis will facilitate investigations of mechanisms, controlling the contribution of identified neural stem cells to the generation of cell diversity and patterning throughout embryonic and postembryonic development of the CNS.

## Materials and Methods

### Parental stocks and embryonic treatment

We combined *elav^C155^*-Gal4 (Lin and Goodman, 1994) with *hs-mFlp5^MH12^* (Shimosako et al., 2014) and crossed virgins of this stock to either UAS-*FB1.1B^260b^* (Shimosako et al., 2014) or to self-made UAS-*FB1.1B^260b^*; *repo*-Gal4 (Sepp et al., 2001) males. Embryos from these crosses were collected for one hour (25°C) on an applejuice-agar petri-dish, provided with yeast. After additional 2.5 hours at 25°C (early cohort; stage 5–8 embryos, according to Campos-Ortega and Hartenstein, 1997) or further 6 hours (late cohort; stage 11 embryos), the petri-dish was sealed with parafilm and heatshocked for 28 minutes in a 37°C-water bath. To stop flipase activity, the parafilm was removed and the petri-dish (with open lid) was set into a water-filled tray inside an incubator (14.5°C). The lid was closed again after half an hour to avoid drying of the embryos and the dish was kept for 16–18 (early cohort) or 20 hours (late cohort) in the 14.5°C-incubator, before it was shifted to 25°C for 5.5 (early cohort) or 2 hours (late cohort) to enhance fluorophore expression. The embryos were dechorionated mechanically by a preparation needle on a double-sided adhesive tape, transferred to a block of applejuice-agar and oriented with their ventral side up under a dissecting fluorescence microscope (Leica MZFLIII). 5–10 embryos were mounted on a cover slip (24×60 mm), desiccated and covered with 10S-Voltalef oil (Lehmann & Voss & Co.) as described before (Prokop and Technau, 1993).

### *In vivo*-documentation of embryonic lineages

The clones were documented in living embryos (stage 16–17) at an inverted confocal microscope (Leica DM TCS SP5) with a 63× glycerol objective. We exclusively used hybrid detection photomultiplier tubes and the same laser settings as described previously (Shimosako et al., 2014). To minimise laser exposition of the embryos, we accelerated scanning by using the bidirectional scanning mode with 400 Hz and two line averages, while using a resolution of 1024×1024 pixels and stacks of 0.8–1.4 µm. To enhance the mTurquoise fluorophore we frequently used a twofold accumulation.

### Larval treatment, fixation and antibody staining

Following documentation, the cover slip was cut into small pieces each carrying a single embryo and set on small applejuice-agar petri-dishes, provided with yeast. Hatching larvae were grown until late L3, when their CNS was prepared as described elsewhere (Bello et al., 2007; Shimosako et al., 2014). For fibre tract staining we used mouse-anti-Nrt (1:10) (Hortsch et al., 1990) (Developmental Studies Hybridoma Bank), which was incubated for several days at 4°C and anti-mouse-Alexa647 (1:500) (Life Technologies) as secondary antibody. Specimens were embedded in Vectashield Mounting Medium (Vector Laboratories) and documented as described above, but scanned at 600 Hz with two frame averages and an additional channel for the Nrt-staining. Most times 70–80 slices were recorded with a thickness between 1–1.5 µm.

### Image analysis

Images were analysed using Volocity Demo 6.1.1 (Perkin Elmer), processed in Adobe Photoshop CS6 and arranged in Adobe Illustrator CS6. The procedure we performed to visualise the embryonic versus the larval origin of cells is described in detail in the first paragraph of the results and in Fig. 1.

The 3D-videos of image stacks were prepared using FluoRender (an interactive tool for multi-channel fluorescence microscopy data visualisation and analysis, SCI, University of Utah).

## Results

The strategy we designed for direct tracing of all embryonic neural cell lineages in the VNC into the late third larval instar requires their *in vivo*-identification in the embryo (Fig. 1A,B). We decided to use the Flybow system, which has been shown to enable clonal analysis in the CNS of living embryos (Hadjieconomou et al., 2011; Fig. 1B). Flybow 1.1B allows *in vivo*-description of clones in three independent hues within the same animal (Shimosako et al., 2014), which offers high efficiency. As a driver line we used *embryonic lethal abnormal vision* (*elav*)-Gal4, which is constantly expressed in all neurons of the CNS and the Peripheral Nervous System (Robinow and White, 1988; Robinow and White, 1991) and is also transiently expressed in embryonic glial cells and NBs (Berger et al., 2007). The default expression of *elav*-Gal4-driven *CD8::GFP* is very useful, as the whole cortex and neuropil are stained. This enables the identification of clones based on the position of cell bodies within the cortex and their projection pattern inside the neuropil. Identification of some lineages was further facilitated by using a combination of *elav*-Gal4 and *reversed polarity* (*repo*)-Gal4 for enhanced labelling of their glial subclone (Xiong et al., 1994). To get complete cell lineages, we induced flipase activity before delamination of the first NBs (early cohort). As we were not able to address all embryonic clones by this early heatshock, we also triggered a late one in a separate group to obtain the missing lineages (see Materials and Methods and Discussion).

Since we are interested in segmental patterning of the CNS, an important goal was to assign each labelled clone to a specific segment along the anterior/posterior (a/p)-axis. This was already possible in the living embryo by counting neuromeres, starting from the most posterior commissure, which is located in anterior A9 (supplementary material Fig. S1). However, *in vivo*-assignment of lineages is difficult in T1 and the gnathal segments, as this region loops towards the brain. Hence, clones within this region have been identified less frequently.

The identified neural cell lineages were reinspected in the late L3 larva. In contrast to *elav*-driven MARCM-clones, which exclusively consist of secondary neurons (Truman et al., 2004), our recombined clones consisted of both secondary and primary sublineages. Generally, primary neurons can be easily distinguished from secondary neurons due to their bigger cell size, their typical location distant from the NB (sometimes even separate from the postembryonic cell cluster), their completely differentiated axonal and dendritic projections and their stronger fluorophore expression (probably due to accumulation). To highlight the primary and secondary parts of a lineage, respectively, we used the following procedure: image stacks of clones selected for presentation were converted from their original colour (Fig. 1C) into grey (Fig. 1D). Secondary cells of a lineage (clustered in direct vicinity to the NB) and their projections [as described by Truman et al. (Truman et al., 2004)] were manually coloured in red. Primary cells and all fibre projections labelled in addition to the postembryonic bundles (thus being considered embryonic) were coloured in green (Fig. 1E,F). As the identification of the complete network of fine arbors was sometimes difficult, since these may cover an enormous area and mix with other cell clones, we only coloured dendritic arbors, which could be traced to the illustrated cell clone. In some figures individual background clones were manually removed for clarity.

**Fig. 2.**
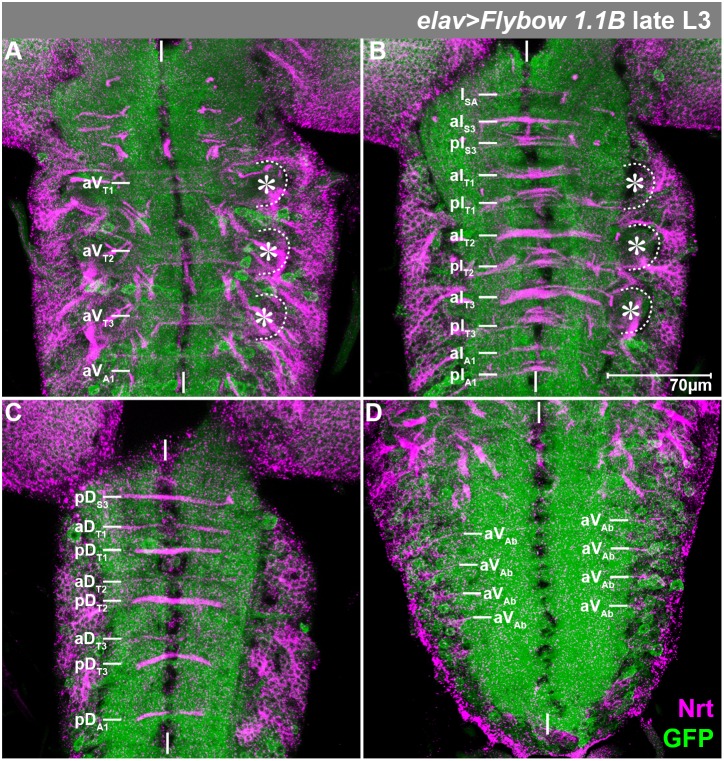
Pattern of Neurotactin-positive fibre bundles. Horizontal view of projections (several sections) through the ventral (A), intermediate (B) and dorsal (C) neuropil of the thorax and through the ventral neuropil of the abdomen (D) of a late L3 larva of the indicated genotype, stained against Nrt (magenta). GFP was recorded only weakly to not interfere with the Nrt-staining. The characteristic Nrt-positive bundles (indicated on the left) serve as landmarks to identify postembryonic cell lineages (see Truman et al., 2004) and corresponding segments. For abbreviations see text. Asterisks mark the leg neuropils, which are additionally highlighted by stippled lines. White vertical bars indicate the midline.

To compare the postembryonic part of the clones to the ones described previously (Truman et al., 2004), we additionally stained all specimens with an antibody against Neurotactin (Nrt; Fig. 1C″), which is exclusively expressed on the surface of immature neurons (de la Escalera et al., 1990) and serves as another criterion for secondary neurons. Furthermore, Nrt-positive neurite bundles provide important landmarks, crossing the midline at characteristic ventral, intermediate or dorsal positions within the anterior and posterior commissure (Truman et al., 2004). The following landmarks were used to determine the precise position of clonal neurite bundles within the neuropil and their segmental affiliation in L3 (see Fig. 2):

aV; broad section of Nrt-positive bundles in the ventral neuropil, running through the anterior commissure of thoracic neuromeres. It is strongly reduced in A1, appears rudimentary in all other abdominal neuromeres and is absent in the gnathal segments (Fig. 2A,D). The posterior commissure does not develop ventral Nrt-positive bundles in any segment.

aI/pI; thick Nrt-positive bundles in the intermediate region of the neuropil, running through the anterior (aI) and posterior commissure (pI) of thoracic neuromeres (Fig. 2B). They are significantly reduced in labial (S3) and A1 neuromeres. There is only one intermediate division of neurite bundles in the anterior part of the subesophageal ganglion (I_SA_, Kuert et al., 2014).

aD/pD; thin Nrt-positive section in the dorsal neuropil, carrying prominent bundles in the posterior commissure (pD) of S3-A1 and less prominent ones in anterior commissure (aD) of T1–T3 (no Nrt-bundles in aD of all other neuromeres; Fig. 2C).

lnp; leg neuropil, a characteristic swelling in the ventral neuropil-region of each thoracic hemineuromere (Fig. 2A,B).

In the following we describe all lineages comprising primary and secondary subclones and assign them to identified NBs and segments (Figs 3–11). 3D-videos of these lineages in L3 are deposited in the supplementary material (supplementary material Movies 1–24). The descriptions are based on 765 induced clones from S3-A9/A10 (supplementary material Table S1). Examples of lineages derived from NBs, which are not reactivated in the larva, are also presented in the supplementary data (supplementary material Figs S2–S5).

**Fig. 3.**
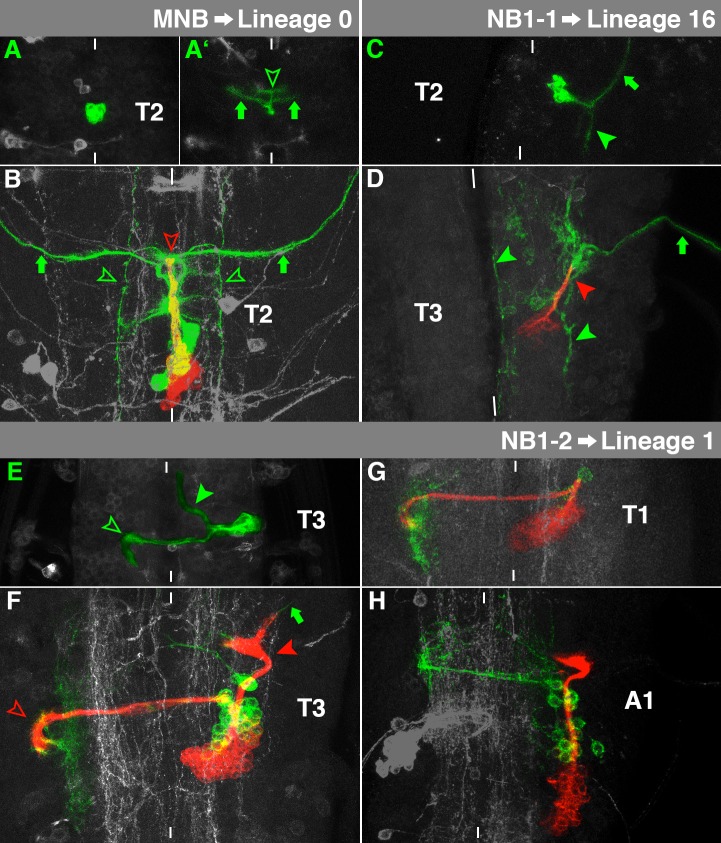
Characteristics of MNB-, NB1-1- and NB1-2-lineage. (A,A′,C,E) Horizontal view (several sections) of the thoracic VNC of living embryos. (B,D,F,G,H) Horizontal view (maximum projection) of the thoracic (B,D,F,G) or anterior abdominal VNC (H) of fixed late third larval instars. In this figure and Figs 4–11, all embryonic images are marked by green, all larval ones by white capital letters. The primary sublineages are illustrated in green and the secondary sublineages in red, which does not reflect their original fluorophore expression (see Materials and Methods). Clone characteristics are highlighted by symbols (green: embryonic origin; red: larval origin; filled arrowhead: ipsilateral projections; hollow arrowhead: contralateral projections, filled arrow: motorprojections, hollow arrow: glial cells) and introduced in the text. Segments are indicated. The background fluorescence of other clones is in grey and allows orientation. White bars indicate the midline. Please see text for morphological details.

**Fig. 4.**
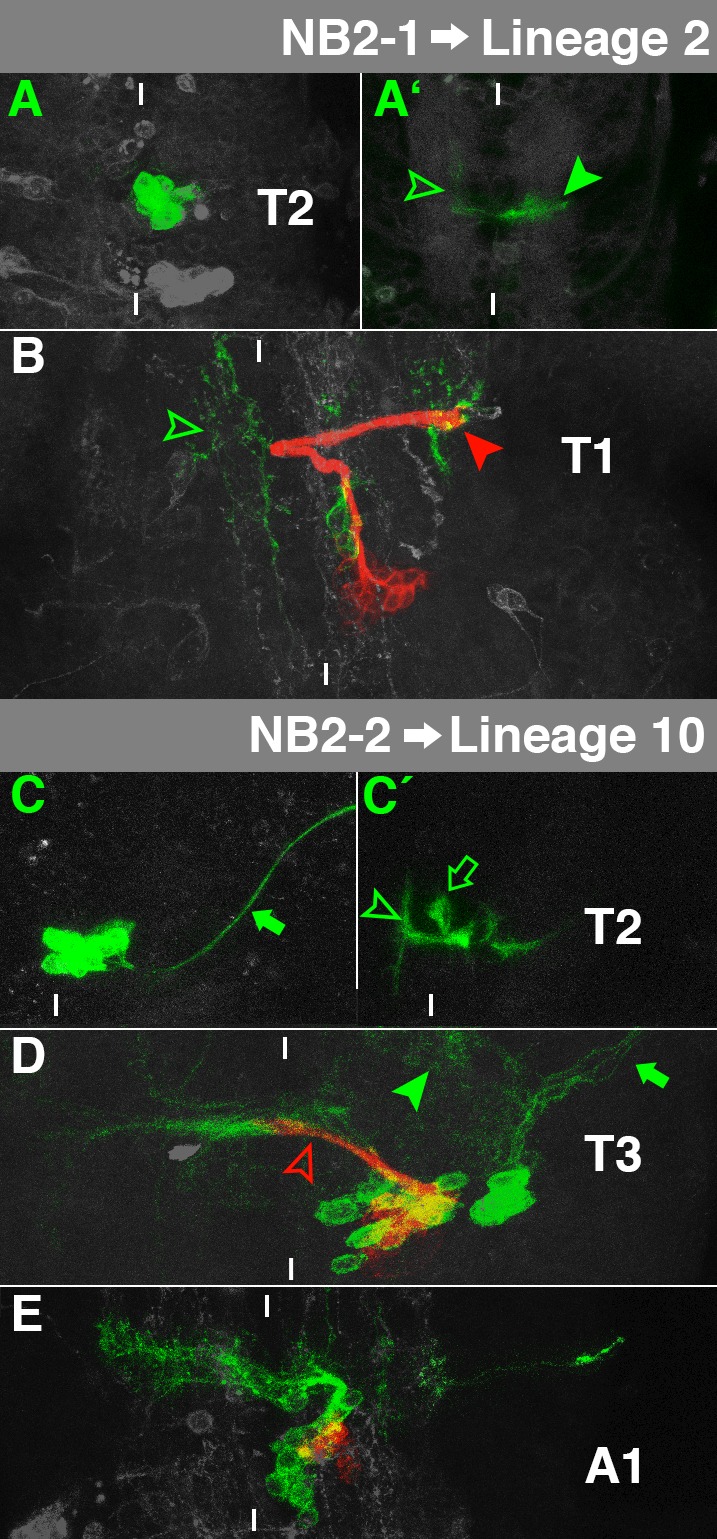
Characteristics of NB2-1- and NB2-2-lineage. For details, see the text and the legend of Fig. 3.

**Fig. 5.**
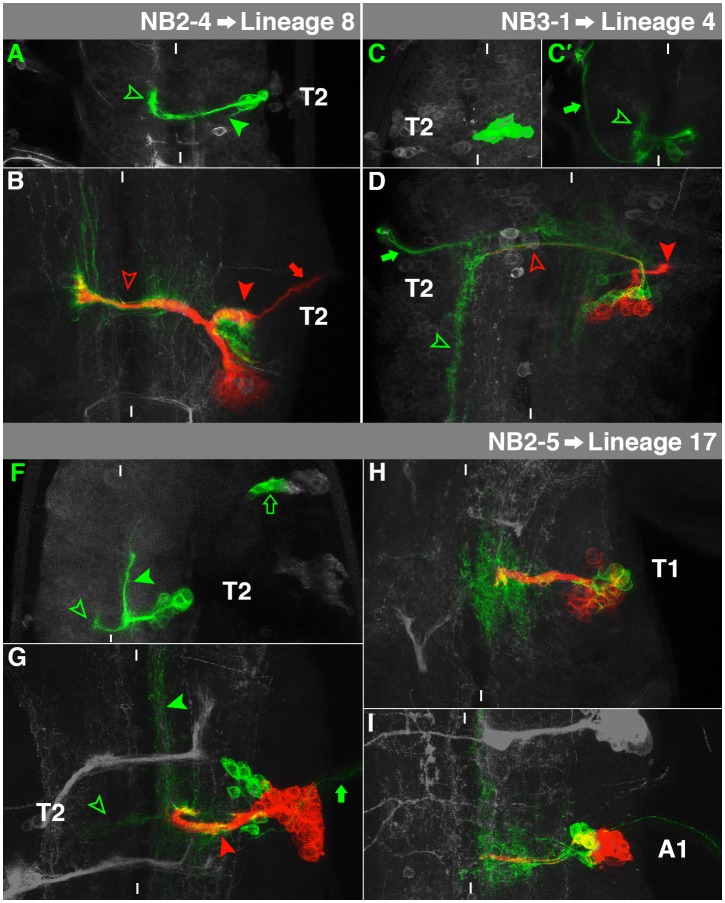
Characteristics of NB2-4-, NB2-5- and NB3-1-lineage. For details, see the text and the legend of Fig. 3.

**Fig. 6.**
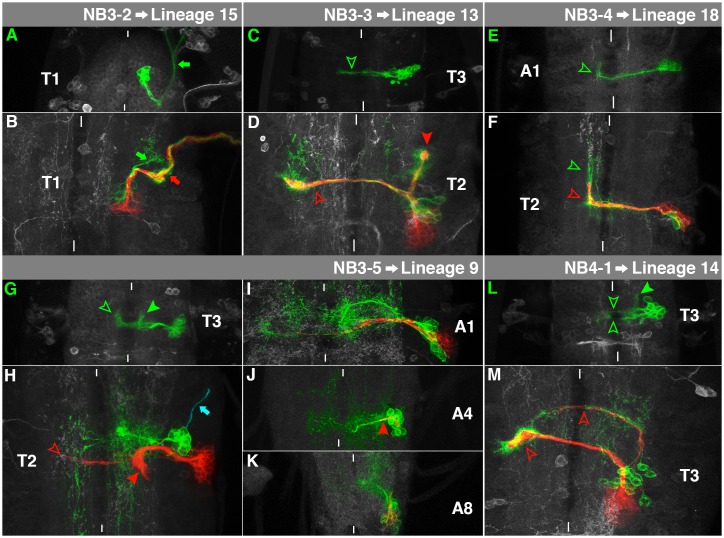
Characteristics of NB3-2-, NB3-3-, NB3-4-, NB3-5- and NB4-1-lineage. For details, see the text and the legend of Fig. 3.

**Fig. 7.**
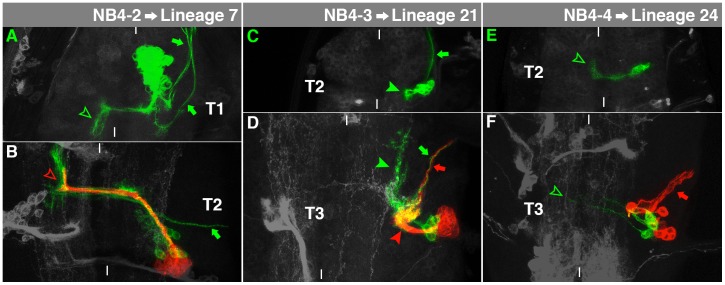
Characteristics of NB4-2-, NB4-3- and NB4-4-lineage. For details, see the text and the legend of Fig. 3.

**Fig. 8.**
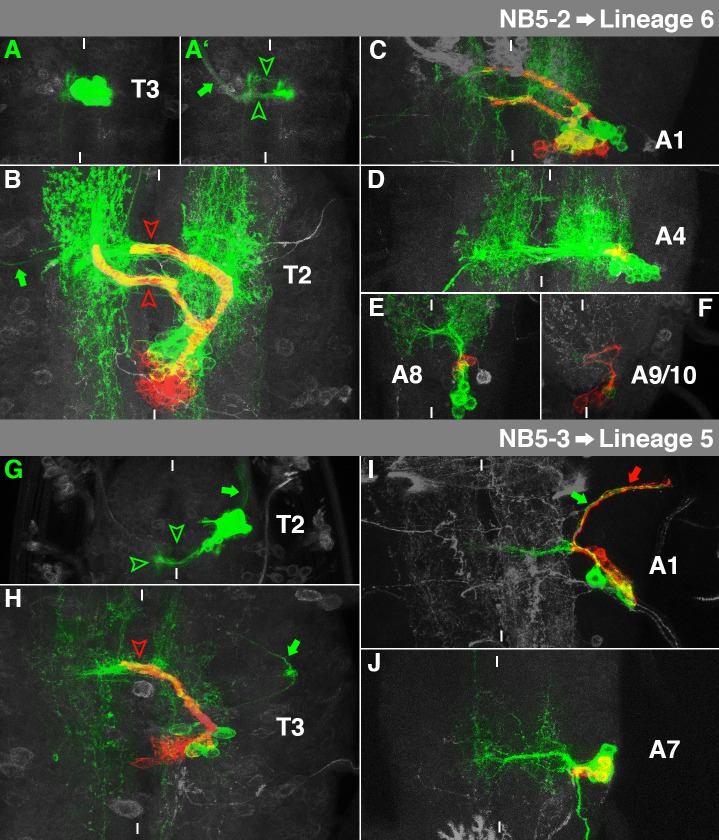
Characteristics of NB5-2- and NB5-3-lineage. For details, see the text and the legend of Fig. 3.

**Fig. 9.**
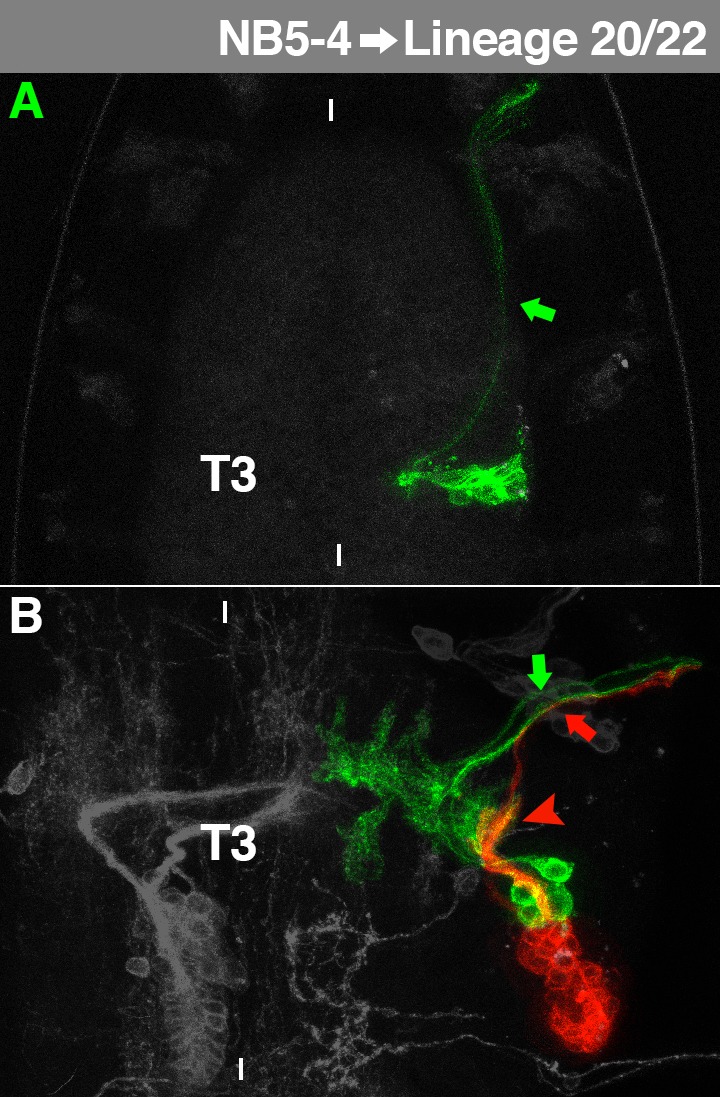
Characteristics of NB5-4-lineage. For details, see the text and the legend of Fig. 3.

**Fig. 10.**
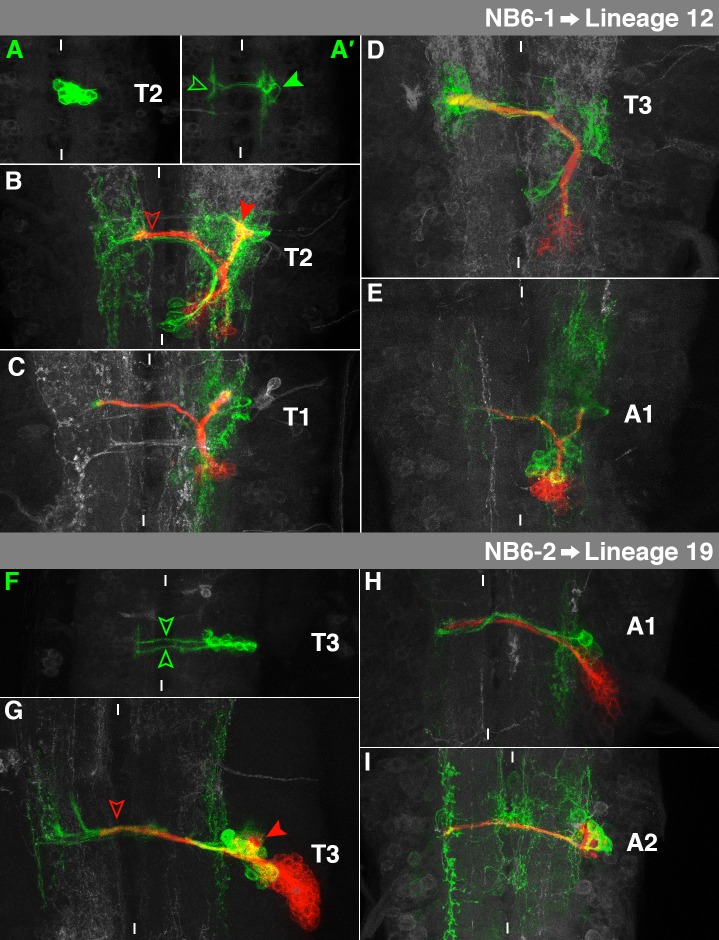
Characteristics of NB6-1- and NB6-2-lineage. For details, see the text and the legend of Fig. 3.

**Fig. 11.**
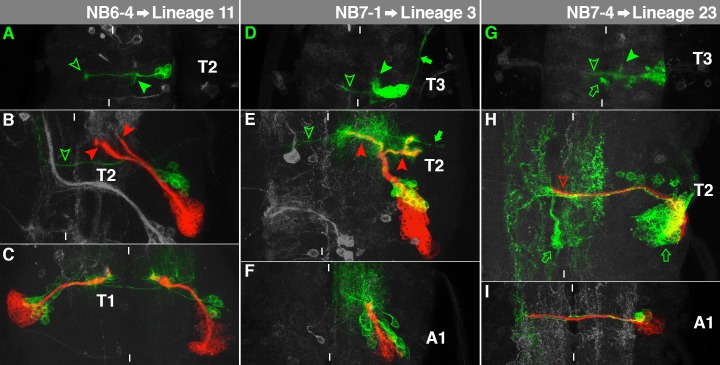
Characteristics of NB6-4-, NB7-1- and NB7-4-lineage. For details, see the text and the legend of Fig. 3.

### Median Neuroblast (MNB)→Lineage 0

The cell clone of MNB consists of inter- and motorneurons, that form an unpaired ventromedial cluster in the late embryo (Fig. 3A). The interneurons project dorsally towards the posterior commissure, where they bend to run anteriorly towards the anterior commissure. They then bifurcate and project laterally (Fig. 3A′, arrowhead). The motorneurons leave the VNC on both sides of the midline (arrows; Bossing and Technau, 1994).

During larval development MNB is reactivated in segments S3-A1. In T2-A1 the secondary neurons form a single medial projection to aI (Fig. 3B, red arrowhead), which corresponds to lineage 0 (Truman et al., 2004). This projection pattern depends on *Ultrabithorax* (*Ubx*)-activity (Marin et al., 2012). Without *Ubx* (S3-T1), the secondary neurons project to pI (Truman et al., 2004). Lineage 0 is Engrailed (En)-positive (Truman et al., 2004), like MNB in the embryo (Doe, 1992). Like in all other ventral clones, the primary neurons are located dorsally to the secondary ones. The embryonic interneurons generate a regular pattern of fine, branched arbors on either side of the midline (Fig. 3B, green arrowheads), while the motorneurons project to aD, where they split and leave the VNC (Fig. 3B, arrows).

### NB1-1→Lineage 16

The embryonic NB1-1 cell lineage comprises a cluster of neurons in a ventromedial position of the cortex (Fig. 3C). The interneurons reveal a posterior projection within the ipsilateral connective (Fig. 3C, arrowhead). The NB1-1 lineage is one of only a few clones, which exhibit no contralateral projections (Bossing et al., 1996b; Udolph et al., 1993). In addition, we sometimes observed the ipsilateral 1-1Ms-motorprojection (Fig. 3C, arrow). The early-born aCC and pCC (Broadus et al., 1995) were never included in our clones, indicating that the recombination occurred after the generation of these progeny cells (see Discussion).

NB1-1 resumes proliferation in all thoracic segments during larval development. The embryonic interneurons exhibit diffuse arborisations only within the ipsilateral connective (Fig. 3D, green arrowheads) and the ipsilateral 1-1Ms shows a prominent efferent projection (Fig. 3D, arrow). The secondary neurons form an ipsilateral, dorsolateral projection (Fig. 3D, red arrowhead) and resemble lineage 16, which supplies local interneurons to the lnp (Truman et al., 2004). Lineage 16 expresses Hb9 and Lim3 (Lacin et al., 2014b), which are already expressed in the embryonic NB1-1 sublineage (Broihier and Skeath, 2002).

### NB1-2→Lineage 1

The embryonic NB1-2 clone can be identified by its ventrolateral interneurons, projecting prominently through the anterior commissure and bending towards posterior in the contralateral connective (Fig. 3E, hollow arrowhead). Furthermore, the NB1-2 lineage displays a pronounced projection towards anterior in the ipsilateral connective (Fig. 3E, filled arrowhead). However, the described projection through the posterior commissure of the next anterior segment was often missing. In addition, we never recovered the TB-neuron, known to be the earliest progeny cell of NB1-2 (Bossing et al., 1996b).

In the larva NB1-2 generates the En-positive lineage 1 (Truman et al., 2004). This lineage underlies a few segment-specific modifications: In T2 and T3 the secondary neurons generate an ipsilateral cone-shaped projection into the lnp of the next anterior segment (Fig. 3F, filled arrowhead). Furthermore, they fasciculate with primary neurons and project through aV, revealing a sickle-like bow towards posterior in the contralateral lnp (Fig. 3F, hollow arrowhead). We sometimes identified a motorneuron (Fig. 3F, arrow), which leaves the VNC from the ipsilateral lnp and seems to have embryonic origin (Schmid et al., 1999). The embryonic and postembryonic ipsilateral projections are missing in T1 (Fig. 3G). In contrast, the ipsilateral bundles are present in A1 (Fig. 3H) where the contralateral bundle only consists of primary neurons, while corresponding secondary neurons normally die due to *Ubx*-expression (Marin et al., 2012).

### NB2-1→Lineage 2

The embryonic cell lineage of NB2-1 can be recognised by its ventromedial cell cluster (Fig. 4A), its thin projection through the dorsal part of the anterior commissure which turns anteriorly after crossing the midline (Fig. 4A′, hollow arrowhead), and its characteristic lateral turn in the dorsal part of the ipsilateral connective (Fig. 4A′, filled arrowhead; Bossing et al., 1996b).

Ipsilateral fascicles of secondary and embryonic neurons mix in the larva. They project dorsally and curve laterally at the level of aD (Fig. 4B, red arrowhead), where the primary neurons arborise extensively. While a few primary neurons cross through aI and arborise in the contralateral connective (Fig. 4B, green arrowhead), secondary neurons do not form a contralateral bundle. The secondary neurons match postembryonic lineage 2, which can be found in all thoracic segments (Truman et al., 2004).

### NB2-2→Lineage 10

The thoracic NB2-2 lineage in the embryo mainly consists of interneurons in a ventromedial cortical position, projecting through the anterior commissure and turning anteriorly and posteriorly after crossing the midline (Fig. 4C,C′, arrowhead). In addition, it contains ipsilateral motorneurons and a subperineurial glia cell (Bossing et al., 1996a; Bossing et al., 1996b), which were all included in our *elav*-driven clones (Fig. 4C,C′, filled and hollow arrow).

The secondary cells of NB2-2 in the larva generate a single projection through aI, which stops shortly after the midline (Fig. 4D, hollow arrowhead). This corresponds to lineage 10 (Truman et al., 2004), which expresses Nkx6, Hb9 and Lim3 (Lacin et al., 2014b). All of these factors are already expressed in the embryonic NB2-2 or parts of its lineage (Broihier et al., 2004; Cheesman et al., 2004; Lacin et al., 2014a). Lineage 10 can be found in all three thoracic segments. The primary cells in L3 appear as two separate clusters: The medial interneurons project together with the secondary ones through aI. Moreover, they form less prominent ipsilateral arborisations (Fig. 4D, filled arrowhead). The lateral cluster is made of ipsilateral motorneurons (Fig. 4D, arrow). The glial cell probably shut down *elav*-expression or died. NB2-2 is also reactivated in A1, although it does not generate postembryonic lineage 10, but only a few Nrt-positive neurons (Fig. 4E). Instead, most of the secondary cells are directly removed by *Ubx*-mediated PCD (Marin et al., 2012).

### NB2-4→Lineage 8

The embryonic cell cluster of NB2-4 is located laterodorsally, just below the level of the neuropil. The interneurons form a projection, which crosses the anterior commissure and displays a prominent curve towards anterior in the contralateral connective (Fig. 5A, hollow arrowhead). Furthermore, they reveal a less pronounced short projection within the ipsilateral connective (Fig. 5A, filled arrowhead). However, our NB2-4 clones never contained a motorneuron (Schmid et al., 1999; Schmidt et al., 1997).

The secondary cells of NB2-4 are placed in a lateral position. They generate a single projection, which bifurcates when reaching the ventral neuropil. The medial branch crosses aI (Fig. 5B, hollow arrowhead), while the lateral branch enters the lnp (Fig. 5B, filled arrowhead). Moreover, the lateral branch contains an ipsilateral motorprojection, leaving the VNC (Fig. 5B, arrow). This matches postembryonic lineage 8 (Truman et al., 2004), which can be found in all thoracic segments. The primary neurons are joined by the secondary ones through aI and reveal complex dendritic arborisations on both sides of the midline. Lineage 8 shares a high degree of *cis*-regulatory modules (CRMs) with lineage 10 (Li et al., 2014), which derives from NB2-2. This is not surprising, as embryonic NB2-2 and NB2-4 reveal almost the same marker gene expression (Broadus et al., 1995).

### NB2-5→Lineage 17

The embryonic cell lineage of NB2-5 consists of interneurons in the dorsal cortex, projecting ipsilaterally towards anterior (Fig. 5F, filled arrowhead) and contralaterally through the anterior commissure (Fig. 5F, hollow arrowhead). Furthermore, we sometimes identified the early-born ipsilateral motorneuron (not shown) and always the two peripheral glia cells (Fig. 5F, arrow; Schmidt et al., 1997).

The secondary neurons form a cluster dorsal to the neuropil. Their axons fasciculate with primary neurons to form a thick, medioventral bundle stalling at aI, not crossing the midline (Fig. 5G, red arrowhead). This corresponds to lineage 17 (Truman et al., 2004). The primary neurons turn sharply at this position to extend anteriorly (Fig. 5G, filled green arrowhead) and their contralateral projection appears very weak (Fig. 5G, hollow arrowhead). The ipsilateral motorneuron can still be found (Fig. 5G, arrow). The glia cells migrated into the periphery and probably shut down *elav*-expression. The secondary NB2-5 lineage can be found in T1 (Fig. 5H) to A1 (Fig. 5I).

### NB3-1→Lineage 4

NB3-1 generates a cluster of ventromedial interneurons (Fig. 5C), which project through the anterior commissure to ramify in the contralateral connective (Fig. 5C′, arrowhead). In a few cases some of the dorsal, contralateral RP-motorneurons were also included in our clones (Fig. 5C′, arrow; Bossing et al., 1996b).

Most secondary cells of NB3-1 form a single projection into the ventral, ipsilateral lnp (Fig. 5D, filled red arrowhead). This resembles postembryonic lineage 4 (Truman et al., 2004), which can be found in T1–T3. However, a few interneurons, which arise in the early larva (Truman et al., 2010), fasciculate with the primary cells to project through aI into the contralateral connective (Fig. 5D, hollow red arrowhead), where the primary neurons extend posteriorly (Fig. 5D, green arrowhead). Primary RP-motorneurons can be found in a dorsomedial position, projecting contralaterally out of the VNC (Fig. 5D, arrow). Lineage 4 expresses Hb9 and Nkx6 (Lacin et al., 2014b). In the embryo Nkx6 is already expressed in NB3-1 (Broihier et al., 2004; Cheesman et al., 2004) and Hb9 in the RP-motorneurons (Broihier and Skeath, 2002).

### NB3-2→Lineage 15

The embryonic NB3-2 cell lineage can be identified by the ventral intermediate location of its cell cluster and pronounced motorneuronal fibres (3-2Mar). They project towards posterior initially, but then turn sharply towards anterior to leave the VNC ipsilaterally (Fig. 6A, arrow). The contralateral projection through the anterior commissure and the 3-2Ms-motorneurons (Bossing et al., 1996b) were absent from our clones.

The secondary sublineage that is formed by larval NB3-2 in thoracic segments exclusively consists of motorneurons. They cross the lnp to leave the VNC ipsilaterally within a thick bundle (Fig. 6B, red arrow), innervating the leg and body wall of the adult fly (Baek and Mann, 2009; Brierley et al., 2012) and match lineage 15 (Truman et al., 2004). Distinct arborisations of the embryonic neurons can be identified (Fig. 6B, green arrow), while their axons fasciculate with the postembryonic motorneurons. Lineage 15 expresses Islet, Lim3 and Nkx6 (Lacin et al., 2014b). Nkx6 is already expressed in the embryonic NB3-2 (Broihier et al., 2004; Cheesman et al., 2004), Islet and Lim3 in embryonic motorneurons (Broihier and Skeath, 2002).

### NB3-3→Lineage 13

The embryonic progeny cells of NB3-3 form an elongated cluster in the ventrolateral cortex, exclusively consisting of interneurons, which generate a projection through the anterior commissure (Fig. 6C, arrowhead). However, the projection reveals no obvious bending towards anterior in the contralateral connective, as described earlier (Schmidt et al., 1997).

The secondary neurons of NB3-3 accompany the primary axons to project dorsally and bifurcate below the neuropil. The thinner medial bundle crosses the midline through aV and spreads in the contralateral lnp (Fig. 6D, hollow arrowhead). The second bundle enters and splays out in the ipsilateral lnp (Fig. 6D, filled arrowhead). This matches postembryonic lineage 13, which is present in all thoracic neuromeres (Truman et al., 2004).

### NB3-4→Lineage 18

The embryonic NB3-4 cell clone is situated in a quite dorsal position of the peripheral cortex. It projects through the anterior commissure and turns sharply towards anterior in the contralateral connective (Fig. 6E, arrowhead; Schmid et al., 1999).

The postembryonic neurons of NB3-4 lie at the level of the intermediate neuropil. Their projections follow the primary ones through aI and bend anteriorly after crossing the midline (Fig. 6F, red arrowhead). This matches lineage 18 (Truman et al., 2004). It can be found in T2-A1, while it is the only lineage, which is absent in T1. The primary neurons have significantly elongated anteriorly in the contralateral connective (Fig. 6F, green arrowhead).

### NB3-5→Lineage 9

The embryonic cell clone of NB3-5 can be found laterally and dorsal to the neuropil. It consists of more than 20 interneurons, sending a thick projection through the anterior commissure and extending bundles towards anterior in the ipsilateral and in the contralateral connective (Fig. 6G, arrowheads; Schmidt et al., 1997).

The bulk of secondary progeny of NB3-5 produce a prominent ipsilateral projection to the intermediate neuropil, not crossing the midline (Fig. 6H, filled arrowhead). Furthermore, they generate a thinner projection through aV (Fig. 6H, hollow arrowhead). This fits lineage 9 (Truman et al., 2004). The primary neurons cross aI and create complex dendritic fields on both sides of the midline. We also recognised a so far undescribed ipsilateral efferent projection, which could be either of embryonic or larval origin (Fig. 6H, arrow). NB3-5 is also reactivated in A1 (Fig. 6I) and in A2–A7 (Fig. 6J), where clone sizes are reduced (supplementary material Figs S7, S8) compared to the thoracic NB3-5 lineage (supplementary material Fig. S6). Its secondary projection (Fig. 6J, arrowhead) reflects the rudimentary aV section of the abdomen (Fig. 2D). Thus, NB3-5 is one of three NBs, which produce a secondary lineage in the posterior abdomen. From its position and the number of progeny cells (up to fourteen) it matches the dorsolateral (dl)-NB (Li et al., 2014; Truman and Bate, 1988). Furthermore, we observed NB3-5 in A8 (Fig. 6K), suggesting that it represents one of the six NBs per side, which become reactivated in the terminal part of the VNC (Taylor and Truman, 1992).

### NB4-1→Lineage 14

Embryonic NB4-1 generates a ventral intermediate cell cluster, exclusively consisting of interneurons, which project through the anterior and posterior commissure of the same segment (Fig. 6L, hollow arrowheads). They also exhibit an anterior projection within the ipsilateral connective (Fig. 6L, filled arrowhead; Bossing et al., 1996b).

In the larva NB4-1 is reactivated in all thoracic neuromeres. Its ventromedial secondary neurons accompany primary projections through aV, anchoring ventrally in the contralateral lnp in a funnel-like manner (Fig. 6M, lower arrowhead). This matches postembryonic lineage 14 (Truman et al., 2004). Only two secondary neurons, which are born in the early larva (Truman et al., 2010), mix with embryonic neurons to project through aD (Fig. 6M, upper arrowhead).

### NB4-2→Lineage 7

Our embryonic NB4-2 clones were located at the lateral edge of the cortex at the level of the ventral neuropil. Interneurons cross the midline through the anterior commissure and reveal a characteristic posterior turn in the contralateral connective (Fig. 7A, arrowhead). The early-born ipsilateral motorneurons (including RP2 and 4-2Mar) were rarely included in our clones (Fig. 7A, arrows; Bossing et al., 1996b; Chu-LaGraff et al., 1995; Udolph et al., 1995).

The secondary neurons of NB4-2 fasciculate with the primary ones to project through aI, curving sharply towards anteriodorsally after reaching the contralateral connective (Fig. 7B, arrowhead). This corresponds to lineage 7, which can be found in S3-A1 (Kuert et al., 2014; Truman et al., 2004). An ipsilateral primary motorprojection can still be observed (Fig. 7B, arrow).

### NB4-3→Lineage 21

The embryonic NB4-3 generates a lateral cell cluster in the ventral cortex. The interneurons reveal little differentiation in the ipsilateral connective (Fig. 7C, arrowhead). The ipsilateral motorneuron leaves the VNC in an anterior direction (Fig. 7C, arrow), while no fibres cross the midline (Schmidt et al., 1997).

The larval subclone of NB4-3 matches lineage 21, which can be found in a ventrolateral position of all thoracic segments (Truman et al., 2004). The secondary neurons send a short ipsilateral projection to enter the medial part of the lnp and spread out extensively (Fig. 7D, red arrowhead). Furthermore, the larval cell clone of NB4-3 contains an ipsilateral motorneuron, which is born during early larval development and innervates the coxa (red arrow, Brierley et al., 2012). It fasciculates with the embryonic motorneuron (Fig. 7D, green arrow). The primary interneurons show extensive ipsilateral projections (Fig. 7D, green arrowhead). Lineage 21 expresses Muscle segment homeobox (Msh; Lacin et al., 2014b), which is expressed in NB4-3 already in the embryo (Isshiki et al., 1997).

### NB4-4→Lineage 24

The embryonic NB4-4 cell clone is characterised by a somewhat round cell cluster adjacent to the neuropil. The clone entirely consists of interneurons which show a projection through the anterior commissure and curve anteriorly in the contralateral connective (Fig. 7E, arrowhead; Schmidt et al., 1997).

The postembryonic subclone of NB4-4 is formed only in the thorax and – in contrast to the primary lineage – consists exclusively of motorneurons, which project into the lnp, from where they turn to leave the VNC ventrally to innervate coxa, trochanter and femur (Fig. 7F, arrow; Baek and Mann, 2009; Brierley et al., 2012). This corresponds to postembryonic lineage 24 (Brown and Truman, 2009). It exhibits the smallest cell number of all larval cell lineages in the thorax (supplementary material Fig. S6). The embryonic interneurons project through aI (Fig. 7F, arrowhead) and spread moderately on both sides of the midline.

### NB5-2→Lineage 6

The embryonic cell cluster of NB5-2 is located ventromedial, close to the midline (Fig. 8A). Most interneurons contribute to a prominent projection through the posterior commissure, bending anteriorly in the contralateral connective (Fig. 8A′, lower arrowhead). The additional projection through the anterior commissure appears rather weak (Fig. 8A′, upper arrowhead). Furthermore, the clone comprises a contralateral motorprojection (Fig. 8A′, arrow; Bossing et al., 1996b).

The secondary neurons of NB5-2 generate a thick bundle, which bifurcates at the level of the ventral neuropil. The medial branch crosses the anterior part of pI to bend anteriodorsally after reaching the contralateral connective (Fig. 8B, lower arrowhead). The lateral branch crosses the midline through pD (Fig. 8B, upper arrowhead). This corresponds to lineage 6, which was found in S3-A1 (Truman et al., 2004). It is En-negative (Truman et al., 2004), but Gooseberry (Gsb)-positive (Li et al., 2014), like NB5-2 in the embryo (Broadus et al., 1995). The embryonically derived interneurons reveal massive arborisations on both sides of the midline (Fig. 8B). The contralateral motorneuron can still be observed (Fig. 8B, arrow). NB5-2 generates one of the biggest cell lineages in the thorax of the late L3 larva, while its size and consequently the secondary bundles are significantly reduced in A1 (Fig. 8C; supplementary material Figs S6, S7). NB5-2 also reveals postembryonic proliferation in the rest of the abdomen (supplementary material Fig. S8), but generates only up to three Nrt-positive cells. The primary subclone exhibits an intermediate and a dorsal bundle through the posterior commissure and the contralateral motorprojection (Fig. 8D). From its position and proliferation behaviour NB5-2 in A2–A7 perfectly matches the ventral medial (vm)-NB (Li et al., 2014; Truman and Bate, 1988). Furthermore, it also contributes secondary neurons to the terminal VNC (Taylor and Truman, 1992): We find mitotically active NB5-2 in A8 (Fig. 8E) and in A9/A10, where it generates up to ten Nrt-positive cells (Fig. 8F).

### NB5-3→Lineage 5

Embryonic NB5-3 clones mainly consist of interneurons in a ventrolateral cortical position. They form a thin projection through the anterior commissure (Fig. 8G, upper arrowhead) and a much stronger bundle through the posterior commissure, from where they turn anteriorly (Fig. 8G, left arrowhead; Bossing et al., 1996b). Moreover, they contain an ipsilateral motorneuron that leaves the VNC anteriorly (Fig. 8G, arrow; Schmidt et al., 1997).

The secondary neurons of NB5-3 project through pI and stop shortly after the midline (Fig. 8H, arrowhead). This matches lineage 5 (Truman et al., 2004). The embryonic interneurons reveal dendritic arborisations on both sides of the midline and the ipsilateral motorneuron can still be identified (Fig. 8H, arrow). The larval subclone of NB5-3 can be found in all thoracic segments and in S3 (not shown), where its expression of Sex combs reduced (Scr) indicates that it belongs to the anterior compartment (Kuert et al., 2014). Its Gsb-expression (Li et al., 2014) matches with NB5-3 in the embryo (Broadus et al., 1995). NB5-3 also proliferates in the abdomen: Similar to NB2-2, we found Nrt-positive NB5-3 progeny in A1, although these are lacking their characteristic projection towards the midline (Fig. 8I). Instead, we observed postembryonic motorneurons within this segment (Fig. 8I, red arrow), accompanying the embryonic motorprojection (Fig. 8I, green arrow). Furthermore, we also noticed secondary neurons of NB5-3 posterior to A1 (Fig. 8J). The position and number of Nrt-positive cells (2–3) match the ventral lateral (vl)-NB (Truman and Bate, 1988).

### NB5-4→Lineage 20/22

The embryonic NB5-4 cell lineage shows a lateral cortical position at the level of the ventral neuropil. It is exclusively composed of motorneurons, projecting medially, but turning when they reach the neuropil to leave the VNC anteriorly (Fig. 9A, arrow; Schmidt et al., 1997).

The larval subclone of NB5-4 can be observed in all thoracic segments. It generates a short projection, which enters the lnp laterally and spreads out extensively (Fig. 9B, arrowhead). Furthermore, the cell cluster contains a postembryonic motorneuron (Fig. 9B, red arrow) innervating the coxa (Brierley et al., 2012). It joins the embryonic motorneurons (Fig. 9B, green arrow) to leave the VNC ipsilaterally. The secondary neurons match lineage 20/22 which are indistinguishable by their morphology (Truman et al., 2004). Both postembryonic lineages express Gsb (Li et al., 2014) and BarH1 (Lacin et al., 2014b), which fits the embryonic expression of the NB5-4 clone (He and Noll, 2013). Moreover, they share a high degree of CRMs (Li et al., 2014). Whenever we observed postembryonic lineage 20/22, we could only identify embryonic NB5-4 as a precursor. Thus, we assume that NB5-4 divides to generate two stem cells with similar potential. This is in accordance with the finding that lineage 20/22 potentially generates transiently amplifying precursors in early larval development (Truman et al., 2010). However, we cannot exclude that NB5-5 (not identified among the labelled clones) is a precursor of lineage 20 or 22, as it expresses many marker genes expressed by NB5-4 in the embryo (Broadus et al., 1995).

### NB6-1→Lineage 12

The embryonic NB6-1 subclone consists exclusively of interneurons located in a ventromedial cortical position (Fig. 10A). They reveal a characteristic circular structure (Fig. 10A′, filled arrowhead) and a prominent projection towards posterior in the ipsilateral connective. In addition, they cross the midline through the posterior commissure and bifurcate in the contralateral connective (Fig. 10A′, hollow arrowhead; Bossing et al., 1996b).

The secondary NB6-1 cell cluster corresponds to lineage 12, which exists in S3-A1 and exhibits significant segment-specific modifications (Truman et al., 2004): In T2 it projects dorsally and splits in the ventral neuropil. One branch crosses the midline through pI (Fig. 10B, hollow arrowhead). The other branch bifurcates again in the intermediate neuropil and both branches remain ipsilateral (Fig. 10B, filled arrowhead). In T1 the second ipsilateral bifurcation was missing in some cases (Fig. 10C). In T3 the whole ipsilateral part of lineage 12 is absent (Fig. 10D) due to *Ubx*-mediated PCD (Marin et al., 2012). In A1 the contralateral and the ipsilateral part are both reduced (Fig. 10E). The embryonic interneurons reveal complex dendritic arborisations on both sides of the midline. Lineage 12 is En-, Gsb- and Dbx-positive (Lacin et al., 2014b; Li et al., 2014; Truman et al., 2004), which matches the marker gene expression of embryonic NB6-1 and its progeny cells (Broadus et al., 1995; Doe, 1992; Lacin et al., 2009).

### NB6-2→Lineage 19

The embryonic interneurons of NB6-2 are located laterally at the level of the ventral neuropil. They send two distinct fascicles through the posterior commissure (Fig. 10F, arrowheads; Bossing et al., 1996b).

The secondary NB6-2 cell cluster is situated in an extreme dorsolateral location and matches lineage 19, which has been found in S3-A1 (Kuert et al., 2014; Truman et al., 2004). The neurons project into the ipsilateral lnp, where they splay out (Fig. 10G, filled arrowhead). Furthermore, they form a projection through pI (Fig. 10G, hollow arrowhead) following the embryonic interneurons. The secondary ipsilateral bundle is missing in A1 due to *Ubx*-dependent PCD (Fig. 10H; Marin et al., 2012). In one case we also found a few secondary NB6-2 progeny cells in A2 (Fig. 10I). Their dorsal position corresponds to the g-NB, which reveals mitotic activity in A2, but not posterior to it (Truman and Bate, 1988). Lineage 19 expresses En, Gsb and Dbx (Lacin et al., 2014b; Li et al., 2014; Truman et al., 2004), all of which are also expressed in the embryonic NB6-2 or a part of its lineage (Broadus et al., 1995; Doe, 1992; Lacin et al., 2009).

### NB6-4→Lineage 11

The neuronal embryonic sublineage of NB6-4 consists of interneurons, which are located at the lateral edge of the cortex, just below the neuropil. They form a projection through the posterior commissure (Fig. 11A, hollow arrowhead). Before reaching the midline the main projection bifurcates and the second branch runs posteriorly along the rim of the ipsilateral connective (Fig. 11A, filled arrowhead; Schmid et al., 1999; Schmidt et al., 1997). The glial progeny cells appeared rarely in our *elav + repo*-induced NB6-4 clones.

The secondary neurons of NB6-4 project medially and split when reaching the neuropil. The ventral arch targets pI, but does not cross the midline (Fig. 11B, lower red arrowhead), the dorsal branch curves dorsally towards anterior (Fig. 11B, upper red arrowhead). However, in T1 the dorsal branch is absent (Fig. 11C, NB6-4 clones on both sides of the midline). These characteristics match lineage 11 (Truman et al., 2004) which is present in S3-T2, while it is the only secondary lineage missing in T3 (and all abdominal segments). Like the embryonic NB6-4 (Doe, 1992) it is an En-positive cluster, belonging to the posterior compartment (Kuert et al., 2014; Truman et al., 2004). The primary neurons cross the midline through the ventral neuropil and curve anteriorly in the contralateral connective (Fig. 11B, green arrowhead).

### NB7-1→Lineage 3

The embryonic NB7-1 lineage is located in a ventral position and extends from the midline to the edge of the cortex. Several U-motorneurons leave the VNC ipsilaterally (Fig. 11D, arrow). The interneurons form a prominent ipsilateral projection towards anterior (Fig. 11D, filled arrowhead) and sometimes a weak contralateral projection through the posterior commissure (Fig. 11D, hollow arrowhead). The described projection through the anterior commissure (Bossing et al., 1996b; Broadus et al., 1995; Goodman and Doe, 1993) has not been identified in our clones.

During larval development NB7-1 gives rise to the largest cell lineage (supplementary material Figs S6, S7). The secondary neurons send out a bundle, which bifurcates in the intermediate region of the neuropil. The lateral branch bends to the ipsilateral lnp, where it generates a so far undescribed bifurcation to infiltrate it completely (Fig. 11E, red right arrowhead). The medial branch targets pD, but does not cross the midline (Fig. 11E, red left arrowhead). This agrees with lineage 3, which is found in S3-A1 (Truman et al., 2004). Due to the absence of lnp in A1, the lateral branch is missing in this segment (Fig. 11F). Lineage 3 expresses En, Gsb and Dbx (Lacin et al., 2014b; Li et al., 2014; Truman et al., 2004). This reflects the embryonic marker gene expression of NB7-1 and its progeny (Broadus et al., 1995; Doe, 1992; Lacin et al., 2009). The ipsilateral primary motorneurons detach from the medial branch in a dorsal position to exit the VNC (Fig. 11E, arrow). A few primary projections cross the midline through pI (Fig. 11E, green arrowhead).

### NB7-4→Lineage 23

The embryonic NB7-4 clone is located in the dorsolateral cortex. The interneurons cross the midline through the posterior commissure (Fig. 11G, hollow arrowhead). Additionally, we found a projection towards anterior in the ipsilateral connective (Fig. 11G, filled arrowhead). Among the glial cells generated by NB7-4 (Schmidt et al., 1997), we sometimes found the channel glia in our *elav*-driven clones (Fig. 11G, arrow) as an unambiguous criterion for NB7-4 identity.

The larval cell cluster of NB7-4 can be found laterally, just ventral to the neuropil. It forms a single bundle through pI (Fig. 11H, arrowhead). This matches lineage 23, which can be found in S3-A1 (Kuert et al., 2014; Truman et al., 2004). On its way through pI the secondary bundle accompanies the embryonic interneurons, which spread on both sides of the midline. Of the embryonic glial cells we identified a channel glia (left arrow) and a subperineurial glia (right arrow). The size and the projections of the clone are significantly reduced in A1 (Fig. 11I). Lineage 23 expresses En (Truman et al., 2004), like NB7-4 in the embryo (Doe, 1992).

## Discussion

Most of the embryonic and postembryonic lineages in the *Drosophila* VNC have previously been characterised and the primary clones have been linked to identified embryonic progenitors (Bossing and Technau, 1994; Bossing et al., 1996b; Schmid et al., 1999; Schmidt et al., 1997; Truman et al., 2004). Furthermore, it has been shown that the secondary lineages also derive from embryonic NBs (Prokop and Technau, 1991). However, technical reasons have so far hampered the assignment of individual secondary lineages to identified embryonic NBs. To establish this link is important, since key features of postembryonic NB-behaviour are already determined in the embryonic neuroectoderm (e.g. Prokop et al., 1998) and many of the factors that specify NBs and their embryonic lineages are known. The approach we have chosen allowed us to close this gap and to identify the embryonic precursor of almost every secondary cell lineage within the VNC.

The *in vivo*-identification of embryonic clones, even upon early heatshock induction, was sometimes challenging, as usually the first-born daughter cells of early delaminating NBs (e.g. aCC and pCC deriving from NB1-1) were not labelled. This and the fact that sufficient numbers of clones from late delaminating NBs (NB2-4, NB3-3, NB4-3, NB4-4, NB5-4, NB6-4) were only obtained after a late heatshock suggests that the recombination is not triggered in the neuroectoderm, but in NBs upon their delamination and often after their first division. Despite this caveat, the embryonic clones exhibited morphological characteristics that allowed their unambiguous identification *in vivo*.

We show how primary and secondary subclones are assembled in each lineage of the late third larval instar (see supplementary material Movies 1–24 for their three-dimensional arrangements). While the secondary lineage typically forms a dense cluster of small cells in the peripheral cortex, the primary subclone consists of rather large, less densely associated cells, which are located closer to the neuropil. The secondary lineage appears to consist of very few neuronal cell types, as they generally form only one or two distinct fibre bundles (Truman et al., 2004). These do not further differentiate before metamorphosis. In contrast, primary lineages generally show a high diversity of cell types (Bossing et al., 1996b; Schmid et al., 1999; Schmidt et al., 1997), displaying fully differentiated, complex axonal and dendritic projections in the late larva. In several of the lineages secondary fibre bundles fasciculate with particular primary projections (e.g. NB1-2, NB2-1, NB2-5, NB3-4). In these cases, the embryonic cells may act as pioneers for the later-born larval neurons.

Each lineage shows a rather high variability in its total cell number and average sizes differ significantly among the various types of lineages within a neuromere (supplementary material Figs S6, S7). Thoracic NBs that produce the largest primary lineages also tend to produce the largest secondary lineages. It needs to be clarified how the different mitotic capabilities among NBs are regulated.

### Segmental specificity of neuroblast reactivation

**Fig. 12.**
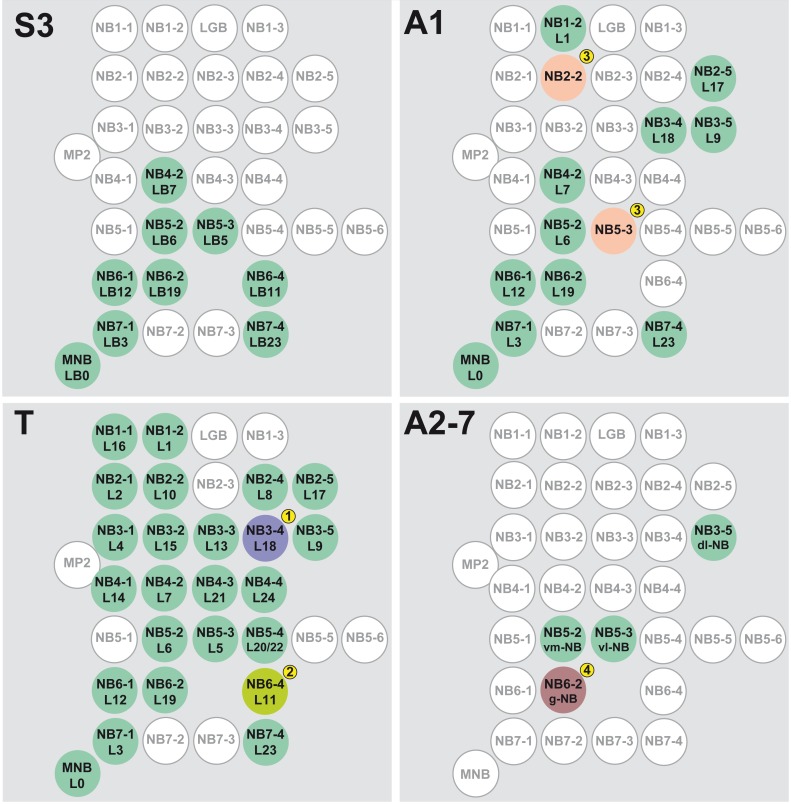
Segment-specific pattern and identities of embryonic neuroblasts, which are reactivated in the larva. Right hemineuromeres of the indicated segments are schematically shown. In each NB its embryonic name (upper row) according to Broadus et al. (Broadus et al., 1995) and Doe (Doe, 1992) and its postembryonic cell lineage (lower row) according to Brown and Truman (Brown and Truman, 2009), Kuert et al. (Kuert et al., 2014) and Truman et al. (Truman et al., 2004) are indicated, except for A2-A7, where the lower row indicates the name of the postembryonic progenitor according to Truman and Bate (Truman and Bate, 1988). NBs which are reactivated in the larva, are highlighted in green; those which are mitotically inactive are white; other colours mark NBs with segment-specific characteristics: (1) Lineage 18 was not identified in T1. (2) Lineage 11 was not identified in T3. (3) NB2-2 and NB5-3 only form a few secondary neurons in A1 and reveal PCD within their lineage. (4) NB6-2 is only reactivated in A2, but not posterior to it.

Which of the 32 (+1 unpaired) embryonic NBs per hemineuromere reenter the cell cycle in the larva depends first of all on the segment in which they are located (summarised in Fig. 12). The complete set of 24 (+1 unpaired) secondary cell lineages (per hemineuromere) within the VNC can be found only in T2 (Brown and Truman, 2009; Truman et al., 2004), which resembles the ground state requiring no *Hox*-input (Lewis, 1978). Only nine NBs are not reactivated in this hemisegment. These preferentially comprise late delaminating NBs (e.g. NB2-3, NB5-1, NB7-3), but there are also exceptions from this tendency (e.g. NB5-6). In contrast, the majority of reactivated NBs are early-born. Thus, in addition to early patterning-genes that specify inter-segmental (*Hox*-genes; reviewed by Technau et al., 2014) and intra-segmental NB-identities (*segment polarity*- and *columnar*-genes; reviewed by Bhat, 1999; Skeath, 1999), the temporal cascade of transcription factors that a NB subsequently expresses (Almeida and Bray, 2005; Isshiki et al., 2001) might influence whether a NB becomes reactivated in the larva or not (see below).

This set of reactivated NBs is reduced by one NB in the first and third thoracic segments, which we identified as NB3-4 (T1) and NB6-4 (T3). For NB2-5 in T1 we confirm the presence of a secondary lineage (see also Kuert et al., 2014; Marin et al., 2012) in contrast to previous suggestions (Lacin et al., 2014b; Truman et al., 2004).

In A1 the number of NBs generating a secondary lineage is drastically reduced to 10 (+1 unpaired) NBs per hemisegment (Truman et al., 2004). However, BrdU pulse chase experiments revealed 12 (+1 unpaired) reactivated NBs in A1 (Truman and Bate, 1988). Accordingly, in addition to 10 (+1) NBs producing rather prominent secondary lineages, we identified two NBs (NB2-2 and NB5-3), which show only a few Nrt-positive progeny cells in the late larva. A1 segmental identity is mediated by *Ubx* (González-Reyes and Morata, 1990) and it is likely that *Ubx* is responsible for the elimination of a subset of NBs in this segment (Bello et al., 2003). The remaining NBs do not express Ubx, but their progeny cells are adapted to segment-specific requirements by Ubx-expression (Marin et al., 2012). The majority of reactivated NBs in A1 are born early with only two exceptions (i.e. MNB and NB3-4).

In the abdomen most NBs undergo apoptosis at the end of embryogenesis (Peterson et al., 2002; White et al., 1994), leaving only three survivors per hemineuromere in A3–A7 that become reactivated in the larva (called dl-, vm-, and vl-NB) and a further one (called g-NB; Truman and Bate, 1988) in A2. We were able to identify these NBs as NB3-5 (dl-), NB5-2 (vm-), NB5-3 (vl-NB) and found evidence that NB6-2 probably refers to the g-NB (Fig. 12), all of which delaminate early (Doe, 1992). The specificity of NBs in this region and their postembryonic proliferation behaviour is determined early in the neuroectoderm under the control of the *Hox*-gene *abdominal-A* (Prokop et al., 1998), which also ends their larval proliferation via PCD (Bello et al., 2003).

Similar to the situation in the posterior abdomen, most NBs in the gnathal neuromeres S1 and S2 undergo apoptosis at the end of embryogenesis. The *Hox*-genes *Deformed* and *Scr* have been shown to prevent the formation of specific postembryonic cell lineages in the subesophageal ganglion (Kuert et al., 2014). Strikingly, in S3 the set of NBs which regains proliferation in the larva, is almost identical to those in A1 (with the exception of NB6-4). It seems that specific groups of serially homologous NBs (same NBs in different segments) are more resistant against *Hox*-mediated prevention of NB-reactivation than others.

Besides the numbers of reactivated NBs, the sizes of serially homologous clones also differ along the a/p-axis: The total number of progeny cells for each NB decreases from thorax, to A1, to posterior abdomen (supplementary material Fig. S8). It is likely that *Hox*-genes are also involved in the reduction of clone sizes in the abdomen, by triggering PCD or by reducing the proliferation rate. This may be mediated by the transcription factor Grainyhead, which has been shown to be required for NBs to produce the number of secondary progeny appropriate to their segmental affiliation (Almeida and Bray, 2005).

## CONCLUSIONS

We established a means to trace the development of identified embryonic NBs through embryonic and postembryonic stages. This allowed us to bridge the gap between primary and secondary lineages in the VNC. Both of these had been previously described in detail, but so far they appeared as separate model systems for larval and adult CNS development, respectively. We traced all lineages into the late larva, but the approach principally allows following labelled clones further into the adult stage (preliminary data) and thus uncovering the full developmental potential of identified embryonic NBs and the contribution of their respective lineages to the formation of functional neural circuits. Our comprehensive analysis also uncovered the complete segmental pattern of those embryonic NBs which become reactivated in the larval VNC, thus revealing the segmental differences in proliferation control of serially homologous NBs and in the composition of their lineages.

The now established link between identified NBs, primary and secondary lineages paves the way for investigations of factors, controlling embryonic development of specific lineages to be extended to their postembryonic development and vice versa.

## Supplementary Material

Supplementary Material
