# Supplementary material for: Bridging the gap between postembryonic cell lineages and identified embryonic neuroblasts in the ventral nerve cord of Drosophila melanogaster
Source: Biol Open. 2015 Mar 27;4(4):420–34. doi: 10.1242/bio.201411072 (PMC4400586; doi:10.1242/bio.201411072)
Supplement: Supplementary Material [file supp_4_4_420__index.html]

Bridging the gap between postembryonic cell lineages and identified embryonic neuroblasts in the ventral nerve cord of Drosophila melanogaster — Bridging the gap between postembryonic cell lineages and identified embryonic neuroblasts in the ventral nerve cord of Drosophila melanogaster — Supplementary Material 

# Bridging the gap between postembryonic cell lineages and identified embryonic neuroblasts in the ventral nerve cord of *Drosophila melanogaster*

## bio.201411072 Supplementary Material

**Files in this Data Supplement:**

- Supplementary Material - Oliver Birkholz et al. doi: 10.1242/bio.201411072
- Movie 1 - **MNB→Lineage 0.**
- Movie 2 - **NB1-1→Lineage 16.**
- Movie 3 - **NB1-2→Lineage 1.**
- Movie 4 - **NB2-1→Lineage 2.**
- Movie 5 - **NB2-2→Lineage 10.**
- Movie 6 - **NB2-4=Lineage 8.**
- Movie 7 - **NB2-5→Lineage 17.**
- Movie 8 - **NB3-1→Lineage 4.**
- Movie 9 - **NB3-2→Lineage 15.**
- Movie 10 - **NB3-3→Lineage 13.**
- Movie 11 - **NB3-4→Lineage 18.**
- Movie 12 - **NB3-5→Lineage 9.**
- Movie 13 - **NB4-1→Lineage 14.**
- Movie 14 - **NB4-2→Lineage 7.**
- Movie 15 - **NB4-3→Lineage 21.**
- Movie 16 - **NB4-4→Lineage 24.**
- Movie 17 - **NB5-2→Lineage 6.**
- Movie 18 - **NB5-3→Lineage 5.**
- Movie 19 - **NB5-4→Lineage 20/22.**
- Movie 20 - **NB6-1→Lineage 12.**
- Movie 21 - **NB6-2→Lineage 19.**
- Movie 22 - **NB6-4→Lineage 11.**
- Movie 23 - **NB7-1→Lineage 3.**
- Movie 24 - **NB7-4→Lineage 23.**
