# Supplementary material for: Bridging the gap between postembryonic cell lineages and identified embryonic neuroblasts in the ventral nerve cord of Drosophila melanogaster
Source: Biol Open. 2015 Mar 27;4(4):420–34. doi: 10.1242/bio.201411072 (PMC4400586; doi:10.1242/bio.201411072)
Supplement: Supplementary Material [file supp_bio.201411072_bio.201411072-s1.pdf]

Biology Open (2015) 000, 1–15 doi:10.1242/bio.201411072

Supplementary Material

Oliver Birkholz et al. doi: 10.1242/bio.201411072

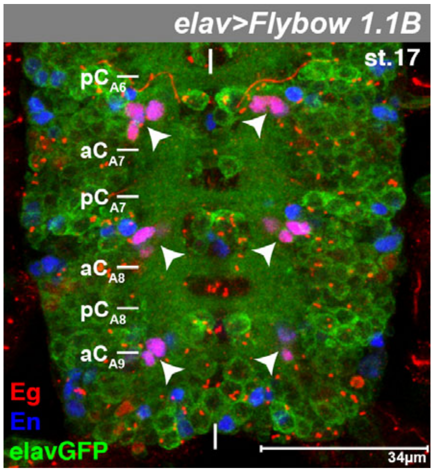

Fig. S1. Segmental assignment of abdominal commissures. Flat preparation (horizontal view, several dorsal sections), showing the posterior abdominal VNC of a stage 17 embryo of the indicated genotype, stained against Engrailed (En, blue), which is expressed in the posterior compartment of each segment, and Eagle (Eg, red). Arrowheads mark the En- and Eg-positive NB7-3 clusters, which are close to the posterior border of each segment. The most posterior NB7-3 cluster is formed in A8 (Birkholz et al., 2013). Anterior (aC) and posterior (pC) commissures of the respective segments are indicated on the left side. The most posterior commissure belongs to the anterior compartment of A9, and was used as a reference for segmental assignment of labelled clones *in vivo*. White vertical bars indicate the midline.

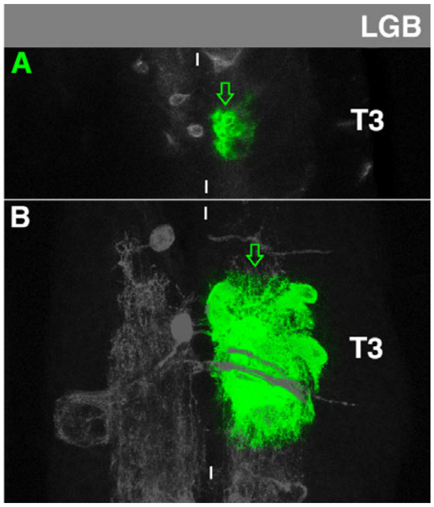

Fig. S2. Characteristics of LGB-lineage. (A) Living stage 17 embryo showing a longitudinal glioblast (LGB) clone comprising several longitudinal glia cells located dorsally on top of the neuropil (arrow; Jacobs et al., 1989; Schmidt et al., 1997). (B) The clone does not reveal further divisions until the late third larval stage, but the cells show significantly extended flat processes (arrow). White vertical bars indicate the midline.

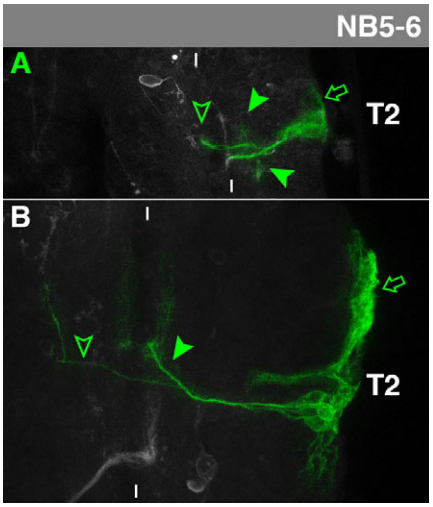

Fig. S3. Characteristics of NB5-6-lineage. (A) The embryonic NB5-6 interneurons in the thorax are located in a dorsal position at the lateral edge of the cortex. They project towards anterior and posterior in the ipsilateral connective (filled arrowheads). Furthermore, they cross the midline through the anterior commissure (hollow arrowhead; Schmidt et al., 1997). The subperineurial glia were included when using the combined *elav/repo*-driver (arrow). (B) NB5-6 does not produce a secondary lineage, which is in line with a previous report showing that it dies in the thorax at the end of embryogenesis (Baumgardt et al., 2009). In the late larva the NB5-6 interneurons are located laterally at the level of the ventral neuropil. They cross the midline through aV and aI to spread in the ipsilateral and somewhat weaker in the contralateral connective (arrowheads). The embryonic subperineurial glial cells enwrap the VNC (arrow). White vertical bars indicate the midline.

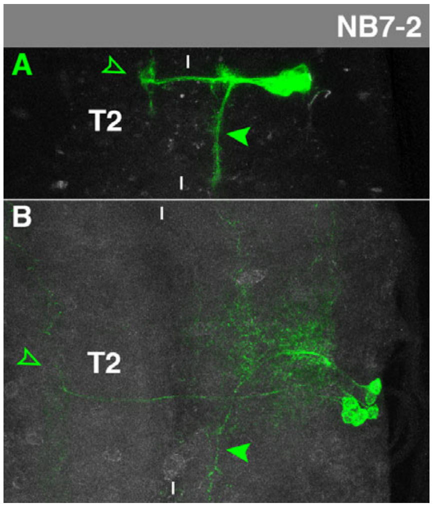

Fig. S4. Characteristics of NB7-2-lineage. (A) The embryonic NB7-2 interneurons are located in an intermediate position at the lateral edge of the cortex. They develop a pronounced ipsilateral projection towards posterior (filled arrowhead). Furthermore, they project contralaterally through the posterior commissure (hollow arrowhead; Bossing et al., 1996). (B) NB7-2 does not produce a secondary lineage. In the late larva the ipsilateral projection extends to the terminal edge of the VNC (filled arrowhead), and there is also massive dendritic arborisation anterior to the clone. The weaker contralateral arbor crosses the midline through the ventral neuropil (hollow arrowhead). White vertical bars indicate the midline.

Biology Open (2015) 000, 1–15 doi:10.1242/bio.201411072

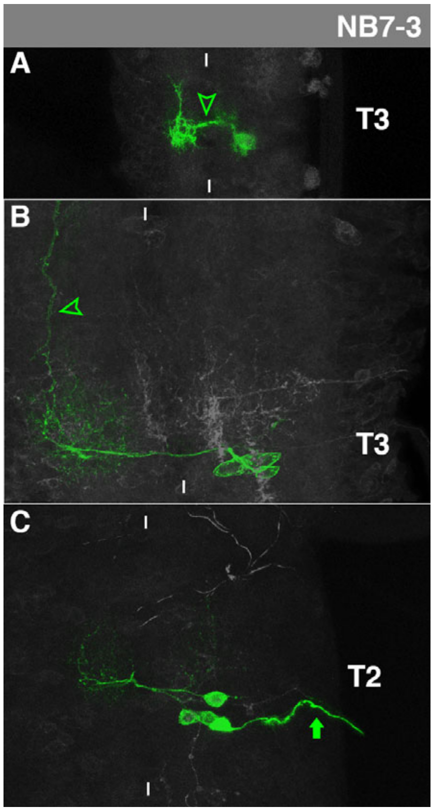

Fig. S5. Characteristics of NB7-3-lineage. (A) The embryonic NB7-3 cell clone in the thorax can be easily identified by its EW1-EW3-interneurons, which form a curved fascicle through the posterior commissure (arrowhead; Bossing et al., 1996). In addition, it contains the GW-neuron (not shown), which survives in T1 and T2, but undergoes PCD in the third thoracic segment (Rogulja-Ortmann et al., 2007). (B) NB7-3 does not produce a secondary lineage, but presumably dies at the end of embryogenesis (see also Karcavich and Doe, 2005; Novotny et al., 2002). The three EW-interneurons cross the midline, turn anteriorly and project into the brain of the L3-larva (arrowhead). (C) In T2 one can identify the surviving ipsilateral GW-neuron, leaving the VNC ventrally (arrow). White vertical bars indicate the midline.

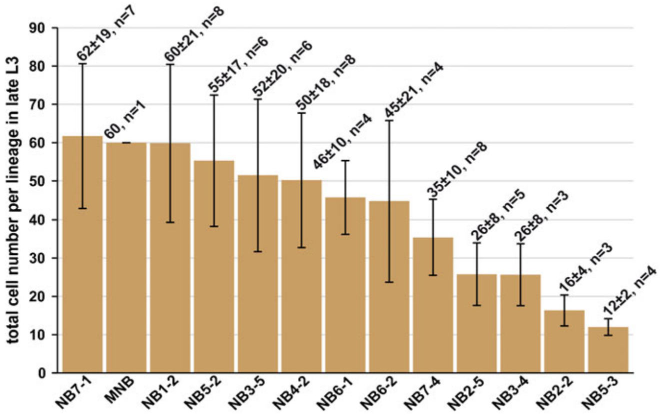

Fig. S7. Total cell number per lineage derived from reactivated neuroblasts in A1. Cell numbers include primary and secondary neurons per clone in late L3 larvae. Error bars represent s.d.; n=number of labelled clones.

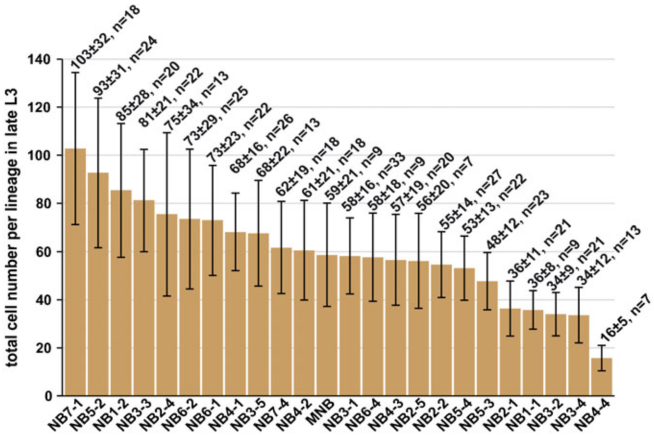

Fig. S6. Total cell number per lineage derived from reactivated neuroblasts in T1–T3. Cell numbers include primary and secondary neurons per clone in late L3 larvae. Error bars represent s.d.; n=number of labelled clones.

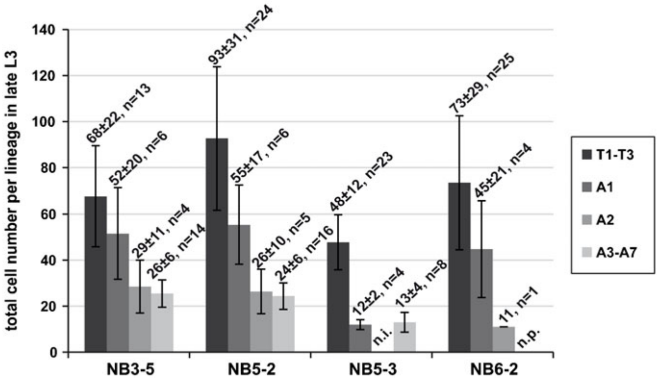

Fig. S8. Intersegmental comparison of total cell number per lineage derived from reactivated neuroblasts in A2–A7 and their serial homologs in A1 and the thorax. Cell numbers include primary and secondary neurons per clone in late L3 larvae. Error bars represent s.d.; n=number of labelled clones; n.i., not identified in the respective segment; n.p., not proliferating in the larva.

Biology Open

Biology Open (2015) 000, 1–15 doi:10.1242/bio.201411072

Movies 1–24. 3D-videos of image stacks from all lineages of reactivated neuroblasts (as indicated) in late L3 (compare Figs 3–11). Views are from posterior (left side) and from dorsal (right side).

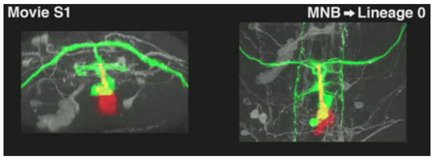

Movie 1. MNB→Lineage 0.

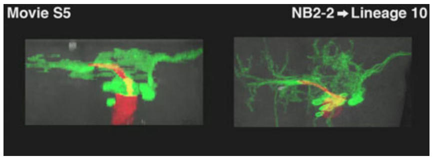

Movie 5. NB2-2→Lineage 10.

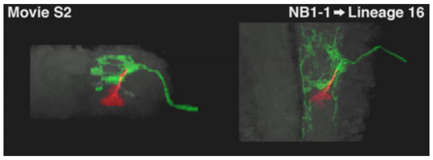

Movie 2. NB1-1→Lineage 16.

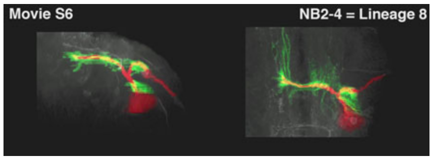

Movie 6. NB2-4=Lineage 8.

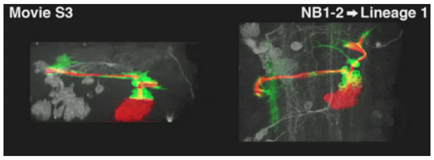

Movie 3. NB1-2→Lineage 1.

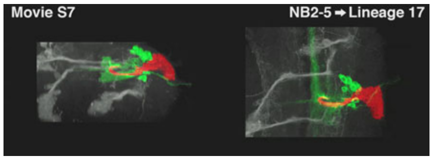

Movie 7. NB2-5→Lineage 17.

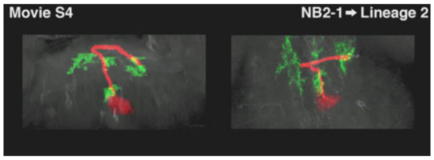

Movie 4. NB2-1→Lineage 2.

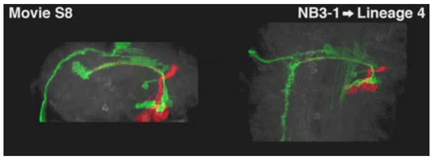

Movie 8. NB3-1→Lineage 4.

Biology Open (2015) 000, 1–15 doi:10.1242/bio.201411072

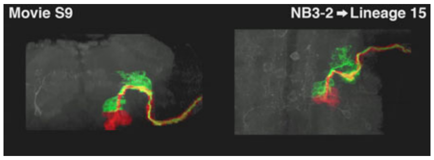

Movie 9. NB3-2→Lineage 15.

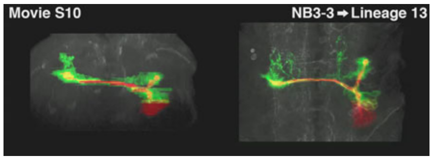

Movie 10. NB3-3→Lineage 13.

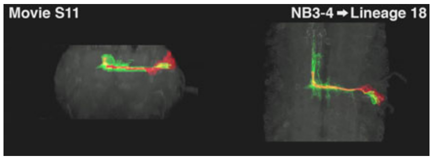

Movie 11. NB3-4→Lineage 18.

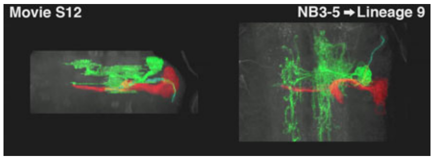

Movie 12. NB3-5→Lineage 9.

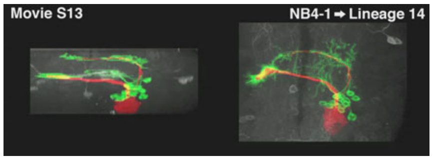

Movie 13. NB4-1→Lineage 14.

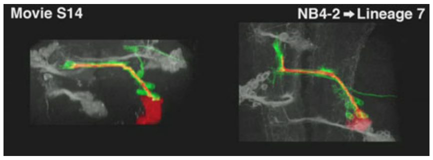

Movie 14. NB4-2→Lineage 7.

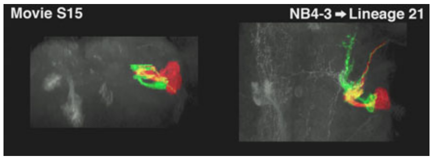

Movie 15. NB4-3→Lineage 21.

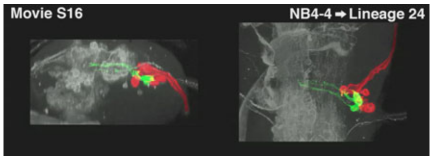

Movie 16. NB4-4→Lineage 24.

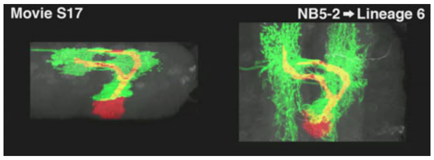

Movie 17. NB5-2→Lineage 6.

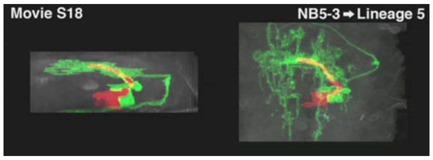

Movie 18. NB5-3→Lineage 5.

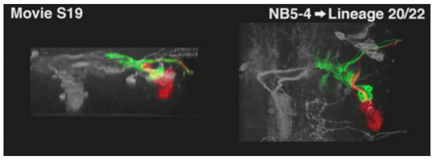

Movie 19. NB5-4→Lineage 20/22.

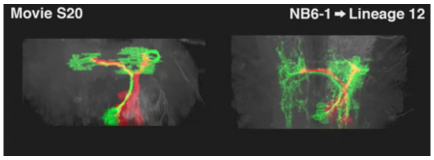

Movie 20. NB6-1→Lineage 12.

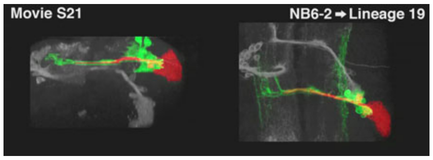

Movie 21. NB6-2→Lineage 19.

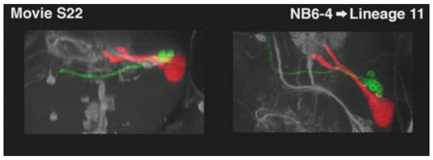

Movie 22. NB6-4→Lineage 11.

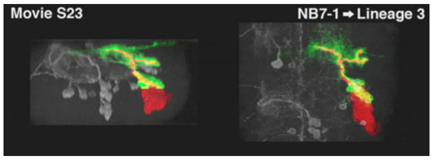

Movie 23. NB7-1→Lineage 3.

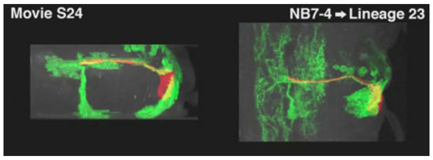

Movie 24. NB7-4→Lineage 23.

Biology Open (2015) 000, 1–15 doi:10.1242/bio.201411072

Table S1. Total amount of analysed clones (n) obtained from reactivated neuroblasts per segment

	S3 (n)	T1 (n)	T2 (n)	T3 (n)	A1 (n)	A2 (n)	A3 (n)	A4 (n)	A5 (n)	A6 (n)	A7 (n)	A8 (n)	A9/10 (n)	Total
NB1-1	n.p.	7	2	5	n.p.	n.p.	n.p.	n.p.	n.p.	n.p.	n.p.	n.p.	n.p.	**14**
NB1-2	n.p.	7	9	13	10	n.p.	n.p.	n.p.	n.p.	n.p.	n.p.	n.p.	n.p.	**39**
NB2-1	n.p.	17	10	6	n.p.	n.p.	n.p.	n.p.	n.p.	n.p.	n.p.	n.p.	n.p.	**33**
NB2-2	n.p.	7	14	12	4	n.p.	n.p.	n.p.	n.p.	n.p.	n.p.	n.p.	n.p.	**37**
NB2-4	n.p.	8	6	5	n.p.	n.p.	n.p.	n.p.	n.p.	n.p.	n.p.	n.p.	n.p.	**19**
NB2-5	n.p.	3	3	6	7	n.p.	n.p.	n.p.	n.p.	n.p.	n.p.	n.p.	n.p.	**19**
NB3-1	n.p.	12	16	20	n.p.	n.p.	n.p.	n.p.	n.p.	n.p.	n.p.	n.p.	n.p.	**48**
NB3-2	n.p.	9	11	6	n.p.	n.p.	n.p.	n.p.	n.p.	n.p.	n.p.	n.p.	n.p.	**26**
NB3-3	n.p.	12	13	7	n.p.	n.p.	n.p.	n.p.	n.p.	n.p.	n.p.	n.p.	n.p.	**32**
NB3-4	n.p.	n.p.	5	9	4	n.p.	n.p.	n.p.	n.p.	n.p.	n.p.	n.p.	n.p.	**18**
NB3-5	n.p.	2	8	7	8	6	3	4	3	2	5	2	0	**50**
NB4-1	n.p.	12	10	13	n.p.	n.p.	n.p.	n.p.	n.p.	n.p.	n.p.	n.p.	n.p.	**35**
NB4-2	1	8	8	10	10	n.p.	n.p.	n.p.	n.p.	n.p.	n.p.	n.p.	n.p.	**37**
NB4-3	n.p.	4	15	12	n.p.	n.p.	n.p.	n.p.	n.p.	n.p.	n.p.	n.p.	n.p.	**31**
NB4-4	n.p.	1	5	1	n.p.	n.p.	n.p.	n.p.	n.p.	n.p.	n.p.	n.p.	n.p.	**7**
NB5-2	3	4	13	16	8	6	1	5	2	7	2	2	3	**70**
NB5-3	3	11	6	9	5	0	8	0	1	0	2	0	0	**45**
NB5-4	n.p.	5	12	12	n.p.	n.p.	n.p.	n.p.	n.p.	n.p.	n.p.	n.p.	n.p.	**29**
NB6-1	3	9	14	9	6	n.p.	n.p.	n.p.	n.p.	n.p.	n.p.	n.p.	n.p.	**41**
NB6-2	0	5	14	10	4	1	n.p.	n.p.	n.p.	n.p.	n.p.	n.p.	n.p.	**34**
NB6-4	1	3	8	n.p.	n.p.	n.p.	n.p.	n.p.	n.p.	n.p.	n.p.	n.p.	n.p.	**12**
NB7-1	2	4	9	10	8	n.p.	n.p.	n.p.	n.p.	n.p.	n.p.	n.p.	n.p.	**33**
NB7-4	6	8	4	10	11	n.p.	n.p.	n.p.	n.p.	n.p.	n.p.	n.p.	n.p.	**39**
MNB	1	1	8	4	3	n.p.	n.p.	n.p.	n.p.	n.p.	n.p.	n.p.	n.p.	**17**
														**765**

n.p., not proliferating in the larva. The increased (n)s compared to supplementary material Figs S6, S7, S8 result from the inclusion of early pupae and larvae, in which cell numbers could not be precisely determined, because of weak fluorophore expression.

Biology Open
